# Extracellular vesicles promote the infection and pathogenicity of Japanese encephalitis virus

**DOI:** 10.1002/jev2.70033

**Published:** 2025-01-09

**Authors:** Junyao Xiong, Ling'en Yang, Xiaowei Nan, Shuo Zhu, Mengxue Yan, Shengxian Xiang, Luping Zhang, Qi Li, Chengjie Yang, Xugang Wang, Ning Wei, Huanchun Chen, Youhui Si, Shengbo Cao, Jing Ye

**Affiliations:** ^1^ National Key Laboratory of Agricultural Microbiology Huazhong Agricultural University Wuhan Hubei China; ^2^ Frontiers Science Center for Animal Breeding and Sustainable Production College of Veterinary Medicine Huazhong Agricultural University Wuhan Hubei China; ^3^ Hubei Hongshan Laboratory Wuhan Hubei China; ^4^ The Cooperative Innovation Center for Sustainable Pig Production Huazhong Agricultural University Wuhan Hubei China

**Keywords:** EVs, JEV, neutralizing antibodies, pathogenicity, tissue barrier

## Abstract

Japanese encephalitis virus (JEV) is a neurotropic zoonotic pathogen that poses a serious threat to public health. Currently, there is no specific therapeutic agent available for JEV infection, primarily due to the complexity of its infection mechanism and pathogenesis. Extracellular vesicles (EVs) have been known to play an important role in viral infection, but their specific functions in JEV infection remain unknown. Here, ultracentrifugation in combination with density gradient centrifugation was conducted to purify EVs from JEV‐infected cells. The purified EVs were found to be infectious, with virions observed inside the EVs. Furthermore, our study showed the formation process of virion‐containing EVs both in vitro and in vivo, which involved the fusion of multivesicular bodies with the cell membrane, leading to the release of virion‐containing intraluminal vesicles into the extracellular space. Further studies revealed that EVs played a crucial role in JEV propagation by facilitating viral entry and assembly‐release. Furthermore, EVs assisted JEV in evading the neutralizing antibodies and promoted viral capability to cross the blood‐brain and placental barriers. Moreover, in vivo experiments demonstrated that EVs were beneficial for JEV infection and pathogenicity. Taken together, our findings highlight the significant contribution of EVs in JEV infection and provide valuable insights into JEV pathogenesis.

## 1 INTRODUCTION

Japanese encephalitis (JE), caused by Japanese encephalitis virus (JEV), is one of the most important mosquito‐borne zoonotic disease in East and Southeast Asia (Endy & Nisalak, 2002; Le Flohic et al., 2013; van den Hurk et al., 2009). Annually, more than 68,000 JE cases are reported, with a fatality rate up to 30%. Additionally, 20%–30% of survivors may suffer from permanent neurologic sequelae, such as inability to speak recurrent seizures, and paralysis (Campbell et al., 2011; Ghosh & Basu, 2009; Misra & Kalita, 2010). Meanwhile, pigs serve as the reservoir and amplifying host for JEV. They develop high levels of viraemia after JEV infection, which can result in orchitis in boars and abortion, stillbirth, and mummified fetus in pregnant sows (Mansfield et al., 2017; Park et al., 2022; van den Hurk et al., 2009; Zheng et al., 2019). Targeting the human central nervous system and pig reproductive system after crossing the respective tissue barriers is a crucial step in the development of diseases during JEV infection.

During the life cycle of flavivirus, virions initially bind to the receptor and attach to the cell membrane, followed by entering the host cell through endocytosis (Nawa et al., 2003; Yang et al., 2013; Zhu et al., 2012). In the early endosome, the acidic environment induces conformational changes of viral E protein, leading to membrane fusion between virions and early endosome (Bressanelli et al., 2004; Modis et al., 2004). The fusion enables the release of viral genome into the cytoplasm. Subsequently, polyproteins are synthesized by ribosomes and cleaved into a series of structural and non‐structural proteins by hydrolases. The viral genome then replicates under the action of RNA replicase and assembles with structural proteins to form immature virions (Uchil & Satchidanandam, 2003). These immature virions undergo further modifications in the endoplasmic reticulum and Golgi apparatus, gradually transforming into mature virions that are eventually released into extracellular space (Lorenz et al., 2003; Yu et al., 2008; Zhang et al., 2003). During JEV infection, viral genome, proteins, immature or mature virions can be selectively packed into multivesicular bodies (MVBs) through endosomal sorting complexes required for transport (ESCRT).

Extracellular vesicles (EVs) are vesicle‐like bodies that are shed from cell membrane or secreted by cells. They have a lipid bilayer structure and are mainly composed of microvesicles and exosomes, with a particle size ranging from 30 to 1000 nm (Jeppesen et al., 2019; Pegtel & Gould, 2019; Raposo & Stoorvogel, 2013; Welsh et al., 2024). EVs are typically generated from ESCRT‐mediated or ceramide‐mediated process (Pegtel & Gould, 2019; Raposo & Stoorvogel, 2013; Zhang et al., 2021). Recent studies have shown a close relationship between EVs and viral infection (Cortes‐Galvez et al., 2023; Li et al., 2024; Martin et al., 2023; Moulin et al., 2023; Reyes‐Ruiz et al., 2020; Zhang et al., 2021). For instance, EVs from the serum of African swine fever virus (ASFV) infected pigs have been observed to selectively recruit viral proteins and porcine host proteins (Montaner‐Tarbes et al., 2019). Similarly, EVs released by herpes simplex virus type 1 (HSV‐1) infected cells have been found to contain viral mRNA and host microRNA, and can be transported from infected cells to uninfected cells (Kalamvoki & Deschamps, 2016; Kalamvoki et al., 2014). Moreover, EVs secreted by cells infected with porcine reproductive and respiratory syndrome virus (PRRSV) (Wang et al., 2018), porcine epidemic diarrhea virus (PEDV) (Chen et al., 2019), classical swine fever virus (CSFV) (Wang et al., 2022), enterovirus 71 (EV71) (Mao et al., 2016), Seneca Valley virus (SVV) (Xu et al., 2020a), Newcastle disease virus (NDV) (Xu et al., 2019), and caprine parainfluenza virus type 3 (CPIV3) (Mao et al., 2020) also can carry viral genome or proteins and contribute to viral escape from neutralizing antibodies (nAbs), resulting in promoting viral infection. Although EVs generally promote viral infection, they also have the ability to inhibit viral replication in a few cases. For example, even though EVs derived from FMDV‐infected cells carry viral genome and proteins, they still exhibit the capability of inhibiting viral replication (Xu et al., 2020b).

In cases of flavivirus infection, EVs released from Zika virus (ZIKV)‐infected *Aedes albopictus* cells (C6/36) have been found to carry viral RNA and envelope protein, and are able to infect and activate both naïve mosquito and mammalian cells (Martínez‐Rojas et al., 2020). Moreover, it has been reported that ZIKV‐infected glioblastoma cells (SNB‐19) secrete distinct infectious EV subpopulations that carry viral E protein and play a role in regulating the release of viral genome and capsid protein (York et al., 2021). During infection with dengue virus serotype 2 (DENV‐2), EVs secreted by C6/36 and *Aedes aegypti* cells (Aag‐2) are shown to mediate the delivery of viral RNA and proteins to naïve human cells (Gold et al., 2020; Vora et al., 2018). As a mosquito‐borne flavivirus in different serogroup from ZIKV and DENV, JEV has a more complex life cycle involving pigs as the intermediate proliferation host, and humans as the terminal host. However, the role of EVs in JEV infection in different species remains unclear.

In this study, EVs derived from JEV‐infected porcine iliac artery endothelial cells (PIEC), Henrietta Lacks (HeLa), and baby hamster kidney (BHK‐21) cells were isolated and purified by ultracentrifugation (UC) and density gradient centrifugation. These EVs were found to be infectious, with virion‐like particles expressing JEV E protein observed inside the EVs. Further study revealed that EVs played a crucial role in promoting viral replication, especially during the entry and assembly‐release process. Additionally, EVs were found to assist JEV in crossing the mouse blood‐brain barrier and porcine placental barrier as demonstrated by using an in vitro transwell model. Moreover, the in vivo experiments demonstrated that EVs promoted viral infection and their entry into the brain in mice. These findings highlight the significant role of EVs in JEV infection and pathogenicity, which enhances our comprehension of the mechanism underlying JEV infection.

## 2 MATERIALS AND METHODS

### 2.1 Cells and viruses

PIEC, HeLa, BHK‐21, and Porcine umbilicus vein endothelial cells (PUVEC) were maintained in DMEM (Sigma Aldrich, USA), supplemented with 10% fetal bovine serum (FBS, Every Green, China), 100 IU/mL penicillin and 100 mg/mL streptomycin at 37°C with 5% CO_2_. Mouse brain microvascular endothelial cell (bEnd.3) and mouse neuroblastoma cells (N2a) were cultured in DMEM (Sigma Aldrich, USA), supplemented with 10% FBM (NSERA, Uruguay), 100 IU/mL penicillin and 100 mg/mL streptomycin at 37°C with 5% CO_2_. The JEV P3 strain (GenBank accession no. U47032.1) was passaged in BHK‐21 cells and stored in our laboratory.

### 2.2 Isolation and purification of EVs

The EVs from JEV‐infected and uninfected cells were prepared and characterized according to the guidelines of MISEV2023 (Welsh et al., 2024). Prior to cell culture, DMEM supplemented with 20% FBS was centrifuged at 150,000 g for 4 h to deplete serum EVs and eventually obtained EV‐free medium. For immunocapture (IC), EVs derived from JEV‐infected cells were first isolated via UC and further purified by IC. Briefly, HeLa cells were infected with JEV at 1 MOI. Following incubation for 1 h, the medium was replaced with EV‐free medium containing 1% FBS, and the supernatants were collected at 36 h postinfection (hpi). The cell cultures were centrifuged at 500 g for 10 min, 2000 g for 10 min and 10,000 g for 30 min to remove cells, dead cells and cell debris, respectively. The supernatants were transferred to SW32Ti centrifugation tubes (Beckman Coulter, USA) and centrifuged at 100,000 g for 90 min, and the pellets were resuspended with PBS (UC‐EVs). Subsequently, 200 µL anti‐CD63 beads (Thermo Fisher Scientific, USA) were mixed with UC‐EVs and incubated for 18–22 h at 4°C. After washing 20 times with isolation buffer (PBS containing 0.1% BSA), the supernatants and CD63‐EVs‐beads (IC‐EVs) were harvested directly and used for western blotting and plaque assay. For the size distribution of EVs, EVs were eluted from the dynabeads using elution buffer (100 nM glycine‐HCl, pH 3.0) and immediately neutralized to pH 7.4 with neutralizing buffer (1 M Tris‐HCl, pH 8.5), followed by dynamic light scattering (DLS).

For density gradient ultracentrifugation (DG), PIEC, HeLa, and BHK‐21 cells cultured as monolayers were infected with JEV at 1, 1, and 0.1 MOI, respectively. Following incubation for 1 h, the medium was replaced with EV‐free medium containing 1% FBS, and supernatants were collected at 36 hpi and subjected to differential centrifugation and UC to isolate EVs (UC‐EVs). The UC‐EVs were then layered onto a discontinuous gradient formed by layering 2.5 mL of 20% sucrose, 2.5 mL of 35% sucrose, 2 mL of 55% sucrose and 2 mL of 70% sucrose in SW41Ti centrifugation tubes (Beckman Coulter, USA). Following centrifugation at 100,000 g for 3 h, fractions of density gradient layers (F1 to F12, each with 1 mL), EVs and EV‐free virions were collected, respectively. Finally, the samples were washed in PBS and pelleted again by UC at 100,000 g for 90 min. The fractions, EVs, and EV‐free virions were resuspended in PBS for subsequent assays. Centrifugation procedures for EV isolation were performed at 4°C. It was important to note that isolated EVs were freshly processed without any frozen step to limit degradation. All relevant data of EV isolation in this study have been submitted to the EV‐TRACK knowledgebase (EV‐TRACK ID: EV240164). The experimental parameters can be reviewed at the following URL: http://evtrack.org/review.php. To access our submission, please use the EV‐TRACK ID (EV240164) and the last name of the first author, Xiong.

### 2.3 Western blotting

Samples were harvested and incubated in lysis buffer (Beyotime Biotechnology, Shanghai, China) containing protease inhibitor for 30 min on ice, and the supernatants were collected after centrifuging at 12,000 rpm for 10 min at 4°C. Followingly, the supernatant can be used for downstream experiment at 95°C for 10 min. The proteins were separated by SDS‐PAGE, transferred to nitrocellulose membrane, incubated with primary antibodies, CD81 (Santa Cruz Biotechnology, USA), TSG101 (Santa Cruz Biotechnology, USA), Alix (Cell signalling, USA) and anti‐JEV M monoclonal antibody (mAb) and E mAb were prepared and preserved in our laboratory. The secondary antibody was labelled goat anti‐mouse IgG (BOSTER, USA). Finally, the proteins were visualized with ECL reagents (Thermo Fisher Scientific, USA) and chemiluminescence system (Tanon, China).

### 2.4 Transmission electron microscope (TEM)

To characterize the morphology of EVs, negative staining was performed. The purified EVs or EV‐free virions were incubated with formvar‐carbon‐coated grids (200 meshes) for 2 min at room temperature (RT). The grids were then washed with PBS and stained with 2% phosphotungstic acid for 2 min. After washing and drying, images were captured by TEM.

For immunoelectron microscope, purified EVs or free‐virus resuspended in PBS were dropped onto parafilm, and a carbon‐coated nickel grid was positioned with the coating side facing the EV droplet for 30 min. Later, the grids were fixed in 2% paraformaldehyde for 10 min. Then the grids were washed 3 times with PBS and blocked for 30 min using 1% bovine serum albumin (BSA). After washing with PBS, the grids were incubated with primary antibody against JEV‐E and CD81 for overnight at 4°C, followed by secondary goat anti‐mouse IgG conjugated with 10‐nm gold (Solarbio, China) for 30 min at RT. After washing 3 times with double distilled water, the grids were stained with 2% phosphotungstic acid for 2 min and processed for electron microscope.

To further explore the process of EV formation in vitro and in vivo, ultrathin sections were performed. In vitro, HeLa cells were infected with JEV at 5 MOI for 12 h. Then the cells were fixed with 2.5% glutaraldehyde for 5 min at RT, and the cells were harvested and centrifuged at 1000 rpm for 5 min. Followingly, the supernatant was discarded and the precipitate was resuspended with 2.5% glutaraldehyde. Finally, the samples were sent to the electron microscope chamber. In vivo, the C57/BL6 mice were inoculated with JEV and sacrificed at 5 days postinfection (dpi). Subsequently, the brain tissues were collected and fixed with 2.5% glutaraldehyde. Eventually, the brain tissues were subjected to ultrathin sections.

### 2.5 Dynamic light scattering

The size distribution of EVs were determined using the Zetasizer system (Malvern Instruments, USA). Briefly, the diluted EV solution (1:100 in PBS with 0.2% BSA) was exposed to a monochromatic laser beam, and the size distribution of EVs was measured by Zetasizer software. The number of replicates for each measurement varied between 13 and 19, depending on the quality of the samples and the default settings of the DLS instrument.

### 2.6 Indirect immunofluorescence

BHK‐21 cells seeded in 24‐well plates were infected with viruses. After 1 h at 37°C, the supernatants were removed, and the cells were washed 3 times with serum‐free DMEM, followed by incubation with 500 µL DMEM containing 1% FBS at 37°C. At the indicated time points, the cells were washed once with PBS, and fixed with paraformaldehyde for 10 min. After washing with PBS, cells were then permeabilized with 0.1% Triton X‐100 in PBS at RT for 10 min. After blocking with 1% BSA in PBS for 30 min, the cells were incubated with anti‐JEV E protein mAb for 1 h. After washing 3 times with PBS, cells were incubated with an Alexa Fluor 488‐conjugated secondary antibody (Invitrogen, USA) for 30 min. Cell nuclei were stained with 4′, 6‐diamidino‐2‐phenylindole (DAPI, Invitrogen, USA) for 10 min. Staining was observed using a fluorescence microscope (Zeiss, Germany).

### 2.7 Labelling and tracing of EVs in vitro

For labelling of EVs, EVs were purified from JEV‐infected PIEC, HeLa, and BHK‐21 cells and labelled with Lipophilic dye Dil (Beyotime, China). Briefly, EVs purified from PIEC, HeLa, and BHK‐21 cells were incubated with Dil for 30 min at RT, then the unlabelled free dyes were removed via sucrose density DG. The labelled EVs were then resuspended in PBS and added to recipient PIEC, HeLa, BHK‐21 cells for uptake experiments. At 30 min, 1 h and 3 hpi, cells were fixed with paraformaldehyde and permeabilized with 0.1% Triton X‐100 in PBS at RT. After blocking with 1% BSA in PBS for 30 min, cells were incubated with anti‐JEV E protein mAb for 1 h. After washing 3 times with PBS, cells were incubated with an Alexa Fluor 488‐conjugated secondary antibody (Invitrogen, USA) for 30 min. Cell nuclei were stained with 4′, 6‐diamidino‐2‐phenylindole (DAPI, Invitrogen, USA) for 10 min. Staining was observed using a confocal microscope (Nikon, Germany).

Dil‐labelled EVs (Dil‐EVs) were incubated with the mouse neuroblastoma cell line N2a to determine whether N2a cells can uptake EVs derived from different cell types. Briefly, Dil‐EVs were incubated with N2a cells for 1 h at 37°C, and then the cells were fixed with paraformaldehyde and permeabilized with 0.1% Triton X‐100. After blocking with 1% BSA, the cells were incubated with an anti‐JEV E protein primary antibody, followed by an Alexa Fluor 488‐conjugated secondary antibody. Cell nuclei were stained with DAPI, and the staining was observed using a confocal microscope. Additionally, to assess the efficacy of N2a cells in the uptake of EVs from different cell types, N2a cells were incubated with EVs derived from PIEC, HeLa, and BHK‐21 cell, containing equal viral RNA copy numbers, for 1 h at 37°C, respectively. Subsequently, the cells were washed 3 times with PBS. Finally, the cells were harvested and viral RNA level were quantified using Taqman quantitative reverse transcription‐PCR (qRT‐PCR).

### 2.8 RNA extraction and quantitative reverse transcription‐PCR

Total RNA was extracted from EVs, EV‐free virions, or cells using TRIzol reagent (Invitrogen, USA), respectively, and the first‐strand cDNA was subsequently synthesized using the ABScript II cDNA First‐Strand Synthesis Kit (ABclonal, China). To determine the RNA copies of JEV in EVs, EV‐free virions, or cells, the Taqman qRT‐PCR was performed with the primers JEV‐E‐F, JEV‐E‐R, and the corresponding probe. A standard curve was generated with a 10‐fold serial dilution of the plasmid encoding the JEV E gene. The relative RNA levels were determined by qRT‐PCR using the SYBR Green mix (ABclonal, China). Primers β‐actin‐F, β‐actin‐R were used for quantification of β‐actin mRNA that served as an internal control. All the primers and probe were listed in Table 1. The qRT‐PCR was performed with the ViiA 7 System (Applied Biosystems, USA).

**TABLE 1:** Primers used in this study.

Primers	Sequences (5′‐3′)	Application
JEV‐C‐F	GGCTTTTATCACGTTCTTCAAGTTT	qRT‐PCR (SYBR Green)
JEV‐C‐R	TGCTTTCCATCGGCCTAAAA	
β‐actin‐F	CACTGCCGCATCCTCTTCCTCCC	
β‐actin‐R	CAATAGTGATGACCTGGCCGT	
IFN β‐F	AACTCCACCAGCAGACAGTG	
IFN β‐R	GGTACCTTTGCACCCT CCAG	
TNF α‐F	CAGGCGGTGCCTATGTCTC	
TNF α‐R	CGATCACCCCGAAGTTCAGTAG	
IL 6‐F	CATGTTCTCTGGGAAATCGTG	
IL 6‐R	TCCAGTTTGGTAGCATCCATC	
CCL 5‐F	TGCCCACGTCAAGGAGTATTTC	
CCL 5‐R	AACCCACTTCTTCTCTGGGTTG	
JEV‐E‐F	TGGTTTCATGACCTCGCTCTC	qRT‐PCR (Taqman)
JEV‐E‐R	CCATGAGGAGTTCTCTGTTTCT	
Probe	CCTGGACGCCCCCTTCGAGCACAGCGT	

### 2.9 Plaque assay

The viruses were serially 10‐fold diluted in DMEM, and the diluted viruses were incubated with BHK‐21 cells in 24‐well plates. After 1 h at 37°C, the supernatants were removed, and the cells were washed 3 times with serum‐free DMEM and overlaid with 1 mL DMEM containing 2% sodium carboxymethyl cellulose and 1% FBS. After incubation for 5 days at 37°C, the cells were fixed with 10% formaldehyde and stained with 0.6% crystal violet solution. Eventually, the visible plaques were counted, and the viral titers (PFU/mL) were calculated.

### 2.10 Viral entry, assembly and release

For viral entry assay, PIEC, HeLa and BHK‐21 cells were incubated EVs and EV‐free virions, containing equal viral RNA copy numbers, at 4°C for 1 h, respectively. After washing 3 times with PBS, the cells were further incubated at 37°C for 1 h to initiate viral entry. Subsequently, the infected cells were stringently washed 3 times with PBS to remove unbound virus and washed for another 3 times with an alkaline high‐salt solution (1 M NaCl and 50 mM sodium bicarbonate [pH 9.5]) to remove surface‐associated virus. Finally, the internalized viruses were quantified by measuring the viral RNA by qRT‐PCR.

For viral assembly and release assay, PIEC, HeLa and BHK‐21 cells were incubated with JEV P3 at 1 MOI for 1 h, and the supernatants were removed and replaced with fresh DMEM containing 1% FBS and DMSO or GW4869. The supernatants or cells were harvested at 24 hpi for plaque assay and qRT‐PCR to determine viral RNA. The assembly efficiency was determined as the ratio of intracellular PFU to intracellular viral RNA copies, while the release efficiency was assessed as the ratio of extracellular PFU to intracellular PFU.

### 2.11 Cell viability assay and GW4869 treatment

Cell viability was measured using Cell Titer‐Glo One Solution Assay kit (Promega, USA) according to the manufacture's instruction. In brief, cells seeded in 96‐well plate were incubated with different concentrations of inhibitors. At 48 hpi, the supernatants were removed and replaced with fresh DMEM. Subsequently, an equal volume of Cell Titer‐Glo reagent was added and shaken at RT for 2 min. The supernatant was subsequently transferred to new cell plates, and the luminescence signal was measured. Finally, the half maximal inhibitory concentration (IC_50_) was calculated. The non‐toxic concentrations of GW4869 (PIEC, 2.5 µM; HeLa, 2.5 µM; BHK‐21, 5 µM) were used for subsequent experiments.

In GW4869 treatment assays, PIEC, HeLa and BHK‐21 cells were infected with JEV at a MOI of 0.01. After 1 h at 37°C, the supernatants were removed and replaced with fresh DMEM containing 1% FBS and DMSO or GW4869. Eventually, the supernatants and cells at 12, 24, 36, and 48 hpi were harvested and titrated, respectively.

### 2.12 Plaque reduction neutralization test (PRNT_50_)

Serum from mice were serially diluted to concentrations of 1:8, 1:16, 1:32, 1:64, and 1:128 with DMEM, and mixed with an equal volume of virus. After incubating at 37°C for 1 h, the mixtures were added to BHK‐21 cells grown in 24‐well plates. The supernatants were removed at 1 hpi, and the cells were washed 3 times with serum‐free DMEM before being overlaid with 1 mL DMEM containing 2% sodium carboxymethyl cellulose and 1% FBS. After incubation for 5 days at 37°C, the cells were fixed with 10% formaldehyde and stained with 0.6% crystal violet solution. Eventually, visible plaques were counted, and the PRNT endpoint titers were expressed as the reciprocal of the serum dilution. The PRNT_50_ titer was calculated based on a 50% reduction in plaque count (Russell et al., 1967).

### 2.13 Neutralization assay in vitro and in vivo

JEV was cultured in BHK‐21 cells and subsequently concentrated through UC. The virus was then inactivated with 0.05% β‐propiolactone. Five‐week‐old mice were immunized with the inactivated virus emulsified with Freund's complete adjuvant. Two weeks later, the mice received a booster immunization with emulsifying with Freund's incomplete adjuvant. Mice inoculated with EVs derived from uninfected cells served as the mock group. Blood was collected 1 week later after booster, and the titers of nAbs in the serum were measured using PRNT50.

In the in vitro neutralization assay, the serially diluted nAbs were incubated with EVs and EV‐free virions, containing equal viral RNA copy numbers, for 1 h at 37°C, respectively. The mixtures were then added to BHK‐21 cells grown on 12‐well plates. After incubation for 24 h at 37°C, the cells were washed 3 times with PBS, and the supernatants and cells were harvested. The viral titers in the supernatants and the intracellular viral RNA levels were quantified by plaque assay and qRT‐PCR, respectively. Eventually, the levels of decreased viral titers and RNA were calculated.

For the in vivo neutralization assay, immunized mice were inoculated intravenously with either EVs or EV‐free virions, containing equal viral RNA copy numbers, 2 weeks after the booster immunization. Mice injected with EVs derived from uninfected cells served as the mock‐infected group. Clinical symptoms and survival rates were monitored daily for 3 weeks. At 3 dpi, blood samples were collected from the mice in each group, and the viral loads in the blood were quantified using Taqman qRT‐PCR.

### 2.14 Transwell culture assay

Transwell culture system (12 mm diameter for 12‐well plate, 0.4 µm polycarbonate membrane; LABSELECT, China) was assembled using bEnd.3 or PUVEC cells. The upper chamber bEnd.3 or PUVEC cells were initially infected with BHK‐21 or PIEC cell‐derived EVs or EV‐free virions containing equal viral RNA copy numbers for 24 h, respectively. The supernatants in the lower chamber were harvested and quantified by Taqman qRT‐PCR.

BHK‐21 or PIEC cells were incubated with JEV at 1 MOI for 1 h, and the supernatants were subsequently replaced by the fresh DMEM containing 1% FBS and DMSO or GW4869. At 36 hpi, the supernatants were harvested, and the viral RNA levels were quantified using Taqman qRT‐PCR. bEnd.3 or PUVEC cells in the upper chamber were then infected with the supernatant containing equal viral RNA copy numbers of JEV from BHK‐21 or PIEC cells, respectively. At 24 hpi, the supernatants in the lower chamber were harvested, and the viral RNA levels were quantified by Taqman qRT‐PCR.

bEnd.3 or PUVEC cells in the upper chamber were infected with JEV for 1 h at 37°C. Then the supernatants were replaced by fresh DMEM containing 1% FBS and DMSO or GW4869. At 24 hpi, the supernatants in the lower chamber were harvested, and the viral RNA levels were quantified by Taqman qRT‐PCR.

bEnd.3 or PUVEC cells in the upper chamber were incubated with EVs derived from PIEC, HeLa and BHK‐21 cells, containing equal viral RNA copy numbers, respectively. At 24 hpi, the supernatants in the lower chamber were harvested, and the viral RNA levels were quantified by Taqman qRT‐PCR.

### 2.15 In vivo EV‐tracing

EVs were incubated with 10 µM DiR (Thermo Fisher Scientific, USA) by incubating for 30 min at RT, and the unlabelled free dyes were removed through centrifugation with 10 kDa ultrafiltration tube. Mice were anesthetized using isoflurane inhalation and subsequently injected with DiR‐labeled EVs either intraperitoneally or intravenously. Mice injected with PBS or free DiR served as control group. In case of intraperitoneal injection, bioluminescence imaging was performed at 6 and 12 h postinjection using an IVIS spectrum (Perkin Elmer, USA), while for tail vein injection, bioluminescence imaging was performed at 4 h postinjection. To determine the distribution of DiR‐labelled EVs across various organs, the brains, hearts, livers, spleens, lungs and kidneys were harvested and imaged separately. The total radiant efficiency was subsequently calculated.

### 2.16 Mouse experiments

All mouse experiments were performed according to protocols approved by the Animal Care and Ethics Committee of Huazhong Agricultural University, under the number HZAUMO‐2023‐0135. All protocols adhered to the Guide for the Care and Use of Laboratory Animals. Three‐week‐old or 5‐week‐old C57BL/6 mice were purchased from Animal Center of Huazhong Agricultural University, housed in an environmentally controlled room, and maintained on standard laboratory food and water throughout the study. To assess the pathogenicity of EVs and EV‐free virions, mice were injected intraperitoneally (i.p.) or intravenously (i.v.) with 100 µL of either EVs or EV‐free virions, containing equal viral RNA copy numbers. Mice in control group were injected with EVs derived from mock‐infected cells. Body weight, clinical symptoms, and survival rates were monitored daily for 4 weeks. At 5 or 8 dpi, mice from each group were sacrificed, and the brain tissues were collected. Ketamine‐xylazine (0.1 mL per 10 g of body weight) was used to anaesthesia the treated mice. The viral loads and mRNA levels of indicated cytokines in the brain tissues were determined by plaque assay and qRT‐PCR, respectively, and the brain tissues were subsequently subjected to hematoxylin and eosin (H&E) and immunohistochemistry (IHC) staining.

### 2.17 Histological and immunohistochemistry staining

For histological staining, brains were fixed in 4% paraformaldehyde overnight at 4°C. At least three brains from different litters from each group were embedded in paraffin for sectioning and subsequently stained with H&E to assess tissue morphology.

For IHC staining, 5 µm thick paraffin sections were placed in 3% H_2_O_2_ for 30 min to quench the endogenous peroxidase activity, and then the sectioned slides were incubated in citrate buffer at 96°C for 30 min for antigen retrieval. After washing with PBS containing 0.1% Tween 20, the sections were blocked in 5% BSA for 1 h and incubated overnight at 4°C with mouse anti‐IBA‐1 primary antibody (Servicebio) diluted in PBS with 0.1% Tween 20. After washing with PBS containing 0.1% Tween 20, the sections were incubated with secondary antibody (horseradish peroxidase‐labelled sheep anti‐mouse IgG, Servicebio) for 45 min. Finally, the slides were developed using the 3,3′‐diaminobenzidine, and hematoxylin was used for counterstaining. All immunohistochemical sections were scanned with a Leica Apero CS2 slide scanning system.

### 2.18 BBB permeability assay

Mice were i.p. with 10 mg of sodium fluorescein dye (NaF, 376 kDa) in 0.1 mL of sterile saline under anaesthesia. After 10 min to allow circulation of the NaF, peripheral blood was collected. Serum (50 µL) was recovered and mixed with an equal volume of 15% trichloroacetic acid (TCA). After centrifugation for 10 min at 10,000 g, the supernatant was recovered and made up to 150 µL by adding 30 µL of 5 M NaOH and 7.5% TCA. At the indicated times, mice were anesthetized and perfused with cold PBS through the left ventricle of the heart to flush out intravascular fluorescein. The brain tissues were homogenized in cold 7.5% TCA and centrifuged for 10 min at 10,000 g to remove insoluble precipitates. After the addition of 30 µL of 5 M NaOH to 120 µL of supernatant, the content of NaF was determined using Spark10M Spectrophotometers (Tecan) with excitation at 485 nm and emission at 530 nm. Standards (125 to 4000 µg/mL) were used to calculate the NaF content of the samples. The permeability of blood‐brain barrier was determined by the ratio (fluorescence intensity in brain tissue/brain tissue weight)/(fluorescence intensity in serum/blood volume). The fluorescence intensity of brain was detected using a small animal in vivo optical imaging system (IVIS Spectrum, Perkin Elmer).

### 2.19 Statistical analysis

All data were analysed using GraphPad Prism 8. The measured values are presented as the mean ± standard deviation (SD) for in vitro experiments and as mean ± standard error of mean (SEM) for in vivo experiments. Statistical significance was analysed using the two‐tailed unpaired Student's *t*‐test, one‐way ANOVA, and two‐way ANOVA for the respective experiments. A difference was considered statistically significant at a *p*‐value < 0.05.

## 3 RESULTS

### 3.1 EVs secreted from JEV‐infected cells display viral infectivity

To determine the optimal condition for the extraction of EVs from cell culture supernatant after JEV infection, cells were infected with JEV and monitored for cytopathic effects (CPE). The supernatant and cells were harvested at 12, 24, 36, and 48 hpi to assess viral infection. The results showed that more than 90% of the cells were infected with JEV (Figure S1A,B) and viral titers peaked at 36 hpi (Figure S1C), but with little cytopathic shedding (Figure S1A). Based on these findings, EVs were extracted from the cell supernatant at 36 hpi.

To investigate the role of EVs in JEV infection, we initially used ultracentrifugation (UC) in combination with IC method (UC‐IC) to purify EVs (referred to as IC‐EVs) from the supernatant of JEV‐infected HeLa cells (Figure S2A). To determine the size distribution of EVs, DLS analysis was performed. We observed that the diameters of purified vesicles ranged from 250 to 500 nm (Figure S2B). Additionally, the presence of EV marker proteins (CD63 and CD81) and viral structural proteins (C, M and E) was detected in the purified vesicles, while the endoplasmic reticulum marker calnexin was not detected (Figure S2C), suggesting that we have successfully obtained purified EVs, which carry the viral structural proteins. Importantly, we demonstrated that the purified IC‐EVs were infectious to BHK‐21 cells by conducting the plaque assay (Figure S2D), indicating that the IC‐EVs may carry infectious virions or full‐length genomic RNA of JEV.

**FIGURE 1:**
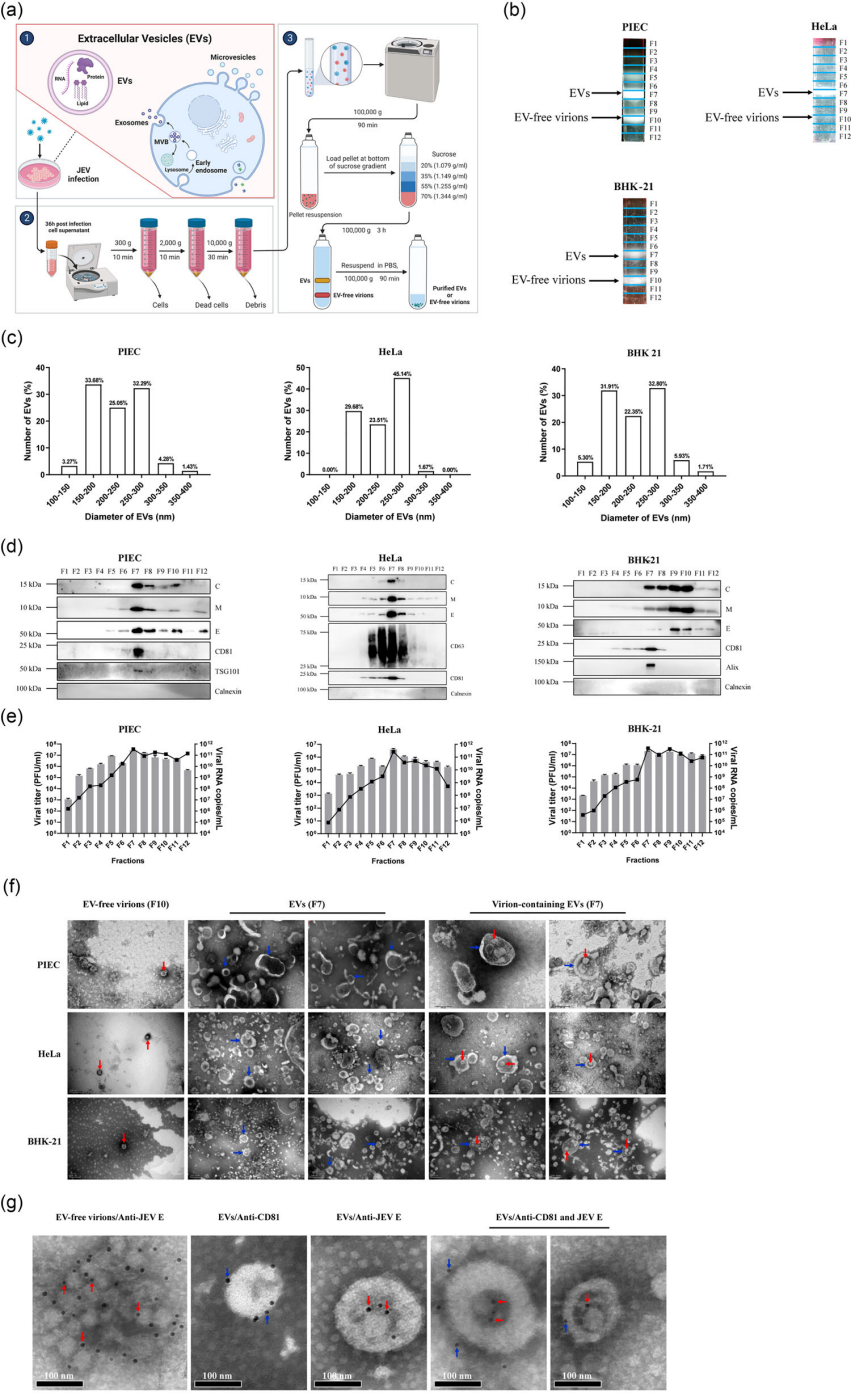
Isolation and characterization of EVs from JEV‐infected cells. (a) Schematic diagram of EVs isolation by ultracentrifugation and further purification by density gradient centrifugation. PIEC, HeLa and BHK‐21 were infected with JEV, and the supernatants were harvested at 36 hpi. The supernatants were centrifugated at 300 g for 10 min, 2000 g for 10 min and 10,000 g for 30 min to deplete cells, dead cells and cell debris, respectively, followed by centrifugated 100,000 g for 90 min. The pellets were resuspended in PBS and loaded on sucrose gradients, followed by centrifugated 100,000 g for 3 h. EVs, EV‐free virions, and fractions (1 mL each) were harvested, followed by washing with PBS at 100,000 g for 90 min. Finally, EVs, EV‐free virions, and fractions were resuspended in PBS. Schematic diagram was created with BioRender (BioRender.com). (b) EVs and EV‐free virions were purified from cell supernatants by sucrose gradient centrifugation. The fraction numbers are indicated in blue. (c) The size distribution of EVs derived from PIEC, HeLa, and BHK‐21 cell. The size diameter of EVs was measured using dynamic light scattering. Data are presented as mean value from three independent experiments. The number of replicates for each measurement varied between 13 and 19, depending on the quality of the samples and the default settings of the dynamic light scattering instrument. (d)–(e) Density gradient centrifugation analysis of fractions. The fractions (1 mL each) were obtained from the top to the bottom of gradient. The expression of viral and EV proteins was detected by Western blotting (d). The representative blots from three independent experiments were shown. Viral titer of each fraction (bar graph) was measured by plaque assay, and viral genome of each fraction (line graph) was quantified by Taqman qRT‐PCR (e). Data are presented as mean ± SD from three independent experiments. (f) TEM observations of negatively stained EVs and EV‐free virions. The EV‐free virions were indicated by red rows, and the EVs were indicated by blue rows. Scale bar: 100 or 200 nm. (g) Immunoelectron microscope observations of EVs and EV‐free virions. The EVs and EV‐free virions were reacted with anti‐CD81 antibody and anti‐JEV E antibody, which were labelled by 10‐nm gold particles, and then observed by electron microscope. The gold‐labelled E proteins on JEV virions were indicated by red rows, while the gold‐labelled CD81 proteins on EVs were indicated by blue rows. Scale bar: 100 nm. BHK‐21, baby hamster kidney; EVs, extracellular vesicles; HeLa, Henrietta Lacks; hpi, hours postinfection; JEV, Japanese encephalitis virus; PIEC, porcine iliac artery endothelial cells; qRT‐PCR, quantitative reverse transcription‐PCR; TEM, transmission electron microscope.

Considering the low yield of EVs by using UC‐IC strategy and the need to isolate EVs from different species of cells, UC combined with density gradient centrifugation (DG) approach (UC‐DG) was adopted as an alternative method to purify EVs from different JEV‐infected cells (Figure 1a). PIEC, HeLa and BHK‐21 cells were infected with JEV, and the cell supernatants were harvested at 36 hpi and subsequently subjected to UC‐DG. Due to the different densities, EV‐free virions and EVs were separated into different fractions (F) (Figure 1b). The results of DLS analysis showed that majority of the EVs derived from these cells exhibited a particle diameter ranging from 150 to 300 nm (Figure 1c). Western blotting analysis using antibodies specific to EVs and JEV proteins revealed that the presence of JEV C, M and E proteins in fraction 10 (F10), while EV marker proteins were not detected. However, both viral proteins and EV markers (CD81 and TSG101 for PIEC cells, CD63 and CD81 for HeLa cells, and Alix and CD81 for BHK‐21 cells) were detected in F7. These findings suggest the presence of EVs and EV‐free virions in F7 and F10, respectively (Figure 1d). We further examined the infectivity of each fraction. Interestingly, we found that all the fractions obtained from JEV‐infected PIEC, HeLa and BHK‐21 cells exhibited infectivity, with the highest infectivity observed in F7 (Figure 1e). Furthermore, we were able to detect the presence of JEV genome in these fractions (Figure 1e). Our study also showed that the level of JEV genome in EVs fraction was approximately 3 times higher than that in EV‐free virions fraction from JEV‐infected PIEC and BHK‐21 cells, while the level of JEV genome in the EVs fraction was approximately 12 times higher than that in the EV‐free virions fraction from JEV‐infected HeLa cells (Figure 1e).

To further verify the characteristics of the purified EVs and EV‐free virions, the TEM was utilized to visualize their morphology. In F7, we observed cup‐shaped morphologies, which are typical EV‐like structures, while enveloped virion‐like particles with a spherical appearance were shown in F10 (Figure 1f). Notably, virion‐like particles were observed within the EVs in F7 (Figure 1a,f), indicating the potential presence of JEV virions within EVs. To further confirm this observation, immunoelectron microscopy was performed. The result revealed that CD81 and JEV E protein were probed in purified EVs and EV‐free virions, respectively (Figure 1g), supporting the successful isolation and purification of EVs and EV‐free virions. Furthermore, JEV E protein was probed on virion‐like particles within the EVs (Figure 1g), which confirmed the presence of JEV virions in the EVs and provided the explanation for their infectivity. Taken together, these findings suggest that the UC‐DG approach is effective in isolating and purifying EVs from cells infected with JEV across various species. These EVs were found to contain virions, genome, and proteins of JEV, which were capable of infecting host cells. All relevant data of EV isolation have been submitted to the EV‐TRACK knowledgebase (EV‐TRACK ID: EV240164), and the EV‐METRIC was calculated to be 63%.

### 3.2 Fusion of virion‐containing MVB with cell membrane leads to the formation of infectious EVs

**FIGURE 2:**
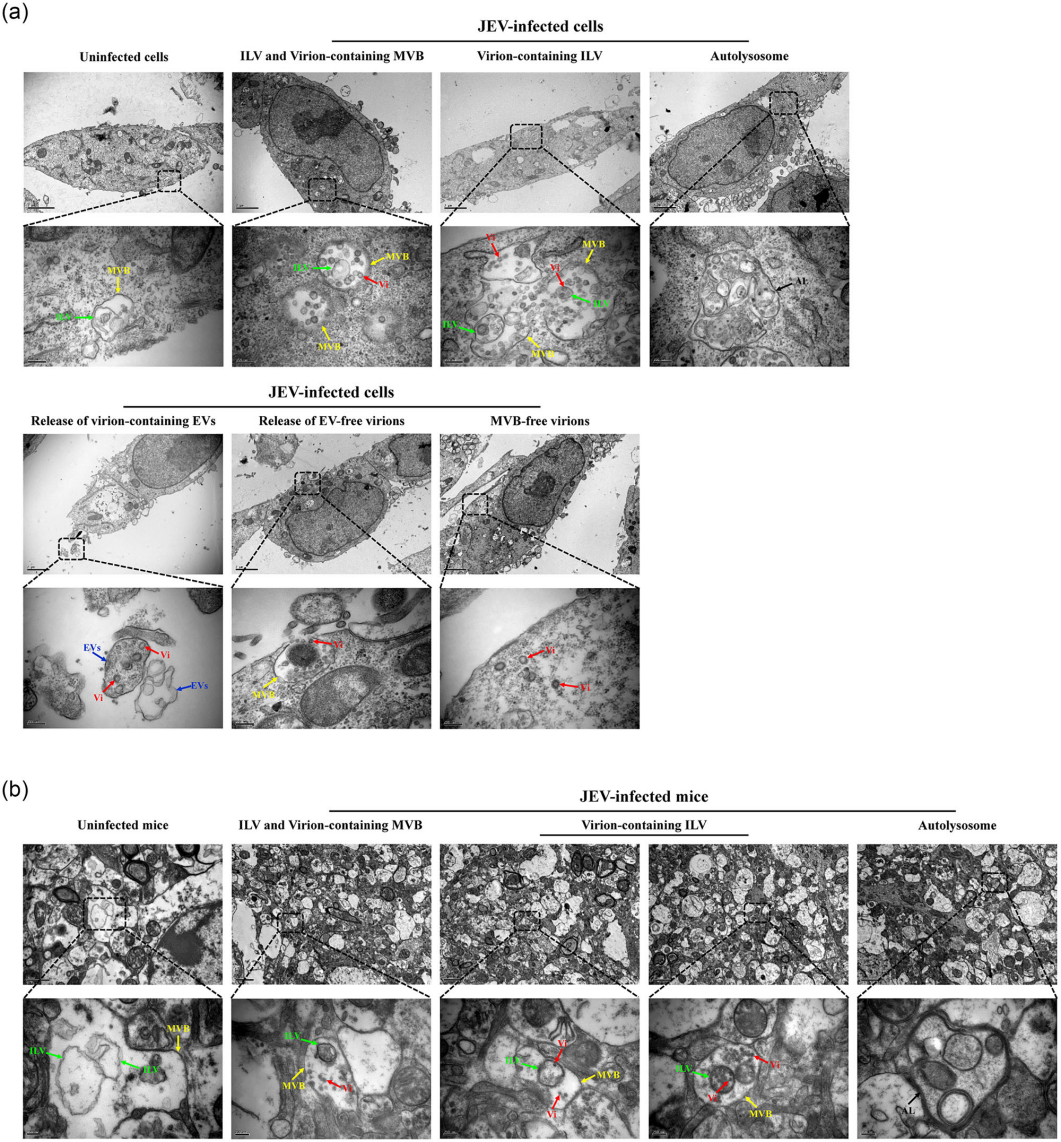
The formation process of EVs in vitro and in vivo. (a) TEM observations of EVs and EV‐free virions in vitro. HeLa cells were infected with JEV at a MOI of 5, followed by fixing with glutaraldehyde at 12 hpi. Samples were sent to the electron microscope chamber and subjected to ultrathin sections. The formation of EVs and EV‐free virions were depicted. The presence of EV‐free virions, EVs, ILV and MVB was indicated by red, blue, green and yellow arrows, respectively. The representative images are shown. Scale bar: upper panel, 2 µm; lower panel, 200 nm. (b) TEM observations of EVs in vivo. C57/BL6 mice were inoculated with JEV and sacrificed at 5 days postinfection (*n* = 3), and the brain tissues were collected for ultrathin sections. The presence of EV‐free virions, EVs, ILV and MVB was indicated by red, blue, green and yellow arrows, respectively. The representative images are shown. Scale bar: upper panel, 2 µm; lower panel, 200 nm. EVs, extracellular vesicles; hpi, hours postinfection; ILV, intraluminal vesicles; JEV, Japanese encephalitis virus; MVB, multivesicular bodies; TEM, transmission electron microscope.

To understand the process of EV formation within cells, HeLa cells were infected with JEV at a MOI of 5. At 12 h post infection, the cells were fixed and ultrathin sections were prepared. In uninfected cells, only MVB and intraluminal vesicles (ILV) were observed, and no virions were found within MVB and ILV (Figure 2a). In JEV‐infected cells, ILV were present within MVB, and virions were found to be enclosed within ILV (Figure 2a). Additionally, the fusion of MVB with cell membrane and the release of virion‐containing ILV into extracellular space were observed (Figure 2a). In addition, a large number of ILV‐free virions were observed in MVB (Figure 2a). Of note, some MVB‐free virions were found inside cells, which may be released in the form of EV‐free virions (Figure 2a). Meanwhile, a portion of MVB were found to undergo autolysosome‐mediated degradation (Figure 2a). These findings suggested that virion‐containing EVs could be released to extracellular space through the fusion of virion‐containing MVB with cell membrane. To further explore the formation process of EVs in vivo, the C57/BL6 mice were intraperitoneally inoculated with JEV, and the brain tissues were subjected to ultrathin sections. We observed the presence of virions and virion‐containing ILV in the MVB of the brain tissue from JEV‐infected mice (Figure 2b), suggesting that JEV can also be released in a form carried by EVs in vivo.

### 3.3 EVs play a critical role in JEV life cycle

**FIGURE 3:**
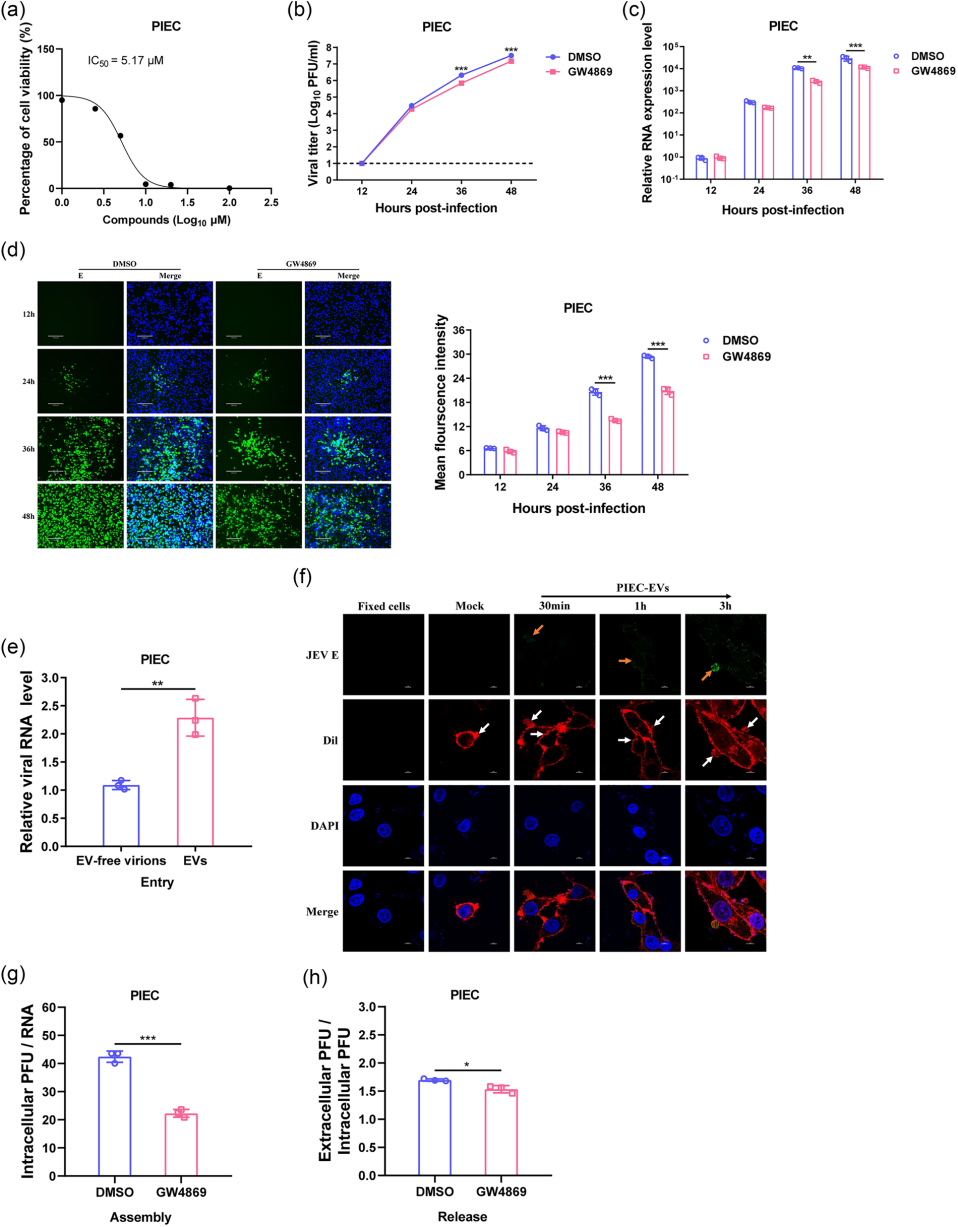
Impact of EVs on JEV multiplication in PIEC cells. (a)–(d) The effect of EVs inhibitor GW4869 on viral multiplication. PIEC were incubated with different concentrations of GW4869. Cell viability was measured using Cell Titer‐Glo One Solution Assay kit, and the IC_50_ of GW4869 was calculated (a). Subsequently, PIEC cells were infected with JEV at a MOI of 0.01, followed by treating with DMSO or GW4869 at 1 hpi. Supernatants and cells were harvested at the indicated time points, and viral titers (b), viral RNA levels (c), and E protein expression (d) were determined using plaque assay, qRT‐PCR and IFA, respectively. Representative images are shown (d, left panel), and the fluorescence intensity was calculated using Image‐Pro Plus from 3 visual fields (d, right panel). The dotted line represents the limit of detection. Scale bar: 130 µm. (e) The effect of EVs on viral entry. PIEC were incubated with EVs and EV‐free virions, containing equal viral RNA copy numbers, at 4°C for 1 h, respectively. After washing with PBS, the cells were further incubated at 37°C for 1 h to initiate viral entry. Then the infected cells were stringently washed 3 times with PBS and an alkaline high‐salt solution to remove surface‐associated virus, and the level of viral RNA entering cell was measured by qRT‐PCR. (f) Visualization of EVs uptake by PIEC cells. EVs purified from PIEC cells were labelled with Dil, and these labelled EVs were incubated with PIEC cells. The cells were then fixed with paraformaldehyde at the indicated time points and blocked with 1% BSA. Subsequently, the cells were incubated with anti‐JEV E mAb, followed by an Alexa Fluor 488‐conjugated secondary antibody and DAPI. Staining was observed by confocal microscope. EVs and JEV E protein were indicated by white and orange arrows, respectively. Representative images from three independent experiments are shown. Scale bar: 5 µm. (g)–(h) The effect of EVs on viral assembly and release. PIEC cells were infected with JEV at a MOI of 1, followed by treating with DMSO or GW4869 at 1 hpi. The supernatants and cells were harvested at 24 hpi for plaque assay and Taqman qRT‐PCR, respectively. The ratio of intracellular viral PFU/RNA copies was used to assess the viral assembly efficiency (e), while the ratio of extracellular PFU/intracellular PFU was employed to evaluate the viral release efficiency (f). Data are presented as mean ± SD from three independent experiments. Statistical analyses were performed using a two‐way ANOVA test followed by the Sidak post‐hoc test (b–(d), or two‐tailed unpaired Student's *t*‐test (e), (g), and (h). ^*^ *p* < 0.05; ^**^ *p* < 0.01; ^***^ *p* < 0.001. BSA, bovine serum albumin; DAPI, 4’, 6‐diamidino‐2‐phenylindole; EVs, extracellular vesicles; hpi, hours postinfection; IFA, Indirect immunofluorescence; JEV, Japanese encephalitis virus; PIEC, porcine iliac artery endothelial cells; qRT‐PCR, quantitative reverse transcription‐PCR.

To investigate the role of EVs in JEV replication, GW4869, a compound known to inhibit the generation of EVs, was utilized to treat JEV‐infected cells. The cytotoxic effects of GW4869 on PIEC, HeLa and BHK‐21 cells were initially assessed by using a luminescence‐based viability assay. The results suggested that the optimal concentration of GW4869 were 2.5 µM for PIEC cells (Figure 3a), 2.5 µM for Hela cells (Figure S3A), and 5 µM for BHK‐21 cells (Figure S3B). Afterwards, PIEC, HeLa and BHK‐21 cells were infected with JEV followed by treatment of GW4869 at these concentrations. Analysis of viral growth kinetics revealed that GW4869 treatment significantly inhibited JEV propagation in PIEC (Figure 3b) and BHK‐21 cells (Figure S3D), suggesting the positive regulatory role of EVs in JEV multiplication. However, GW4869 had an opposite effect on JEV propagation in HeLa cells (Figure S3C). Consistently, lower levels of viral RNA were detected in JEV‐infected cells treated with GW4869 at most time points compared to those treated with DMSO (Figure 3c, Figure S3E,F). Additionally, reductions in viral protein levels were observed in GW4869‐treated cells compared to DMSO‐treated cells (Figure 3d, Figure S3G,H). These findings indicated that EVs production may have varying effects on JEV life cycle in different cell types.

As it has been reported that EVs can mediate viral invasion in a receptor‐independent way, the impact of EVs on the initial entry step of JEV infection was investigated. Briefly, PIEC, HeLa and BHK‐21 cells were incubated with EVs and EV‐free virions, containing equal viral RNA copy numbers, at 4°C for 1 h, respectively, and viral entry was then initiated by incubation for another 1 h at 37°C. The viral RNA levels were subsequently determined by qRT‐PCR. The results showed that viral RNA level in infectious EVs‐treated cells was higher than that in cells infected with EV‐free virions, indicating that EVs promoted viral entry process (Figure 3E, Figure S4A,B). To further verify that virion‐containing EVs could mediate the invasion of JEV, lipophilic dye Dil was used to label EVs. PIEC, HeLa and BHK‐21 cells were incubated with Dil‐EVs and the signal of Dil and JEV E protein were observed. The results showed a time‐dependent increase in the abundance of Dil signal and viral E protein on the cell surface and in the cytoplasm of PIEC, HeLa and BHK‐21 cells (Figure 3f, Figure S4C,D), suggesting that the cells took up the virion‐containing EVs. In addition, we observed that if the cells were fixed by paraformaldehyde, they remained negative for Dil signal (Figure 3f, Figure S4C,D), indicating that the uptake of EVs was an active process rather than passive transfer of fluorescent label.

JEV is a neurotropic virus known to induce significant damage to brain tissue. Upon entering the brain, the virus primarily targets neuronal cells and causes extensive neuronal death, which can ultimately result in mortality. Therefore, we next investigated whether EVs derived from different cell types can be internalized by neurons. To this end, Dil‐EVs derived from PIEC, HeLa and BHK‐21 cells were incubated with the mouse neuroblastoma cell line N2a, respectively, and the uptake of labelled EVs by N2a was observed. Additionally, to assess the efficacy of N2a cells in uptake of EVs, N2a cells were incubated with EVs derived from various cell lines at equivalent viral RNA copy numbers, followed by assessing the viral RNA levels in the cells at 1 hpi. The results showed that N2a cells are capable of internalizing EVs derived from different cell types (Figure S4E), but there are differences in uptake efficiency, with the highest uptake observed for EVs from BHK‐21 cells (Figure S4F).

Considering the important role of MVB and ILV in viral assembly and release, it is worthwhile to explore whether the formation of EVs contributes to the regulation of JEV assembly and release. PIEC, HeLa and BHK‐21 cells were infected with JEV followed by treating with GW4869. The supernatants and cells were harvested at 24 hpi, and viral titers in the supernatants and intracellular viral RNA abundance were measured by plaque assay and Taqman qRT‐PCR, respectively. To measure viral assembly efficiency, the intracellular viral PFU/RNA copies ratios were calculated. The results showed that GW4869 treatment reduced the viral PFU/RNA copies ratios in PIEC, HeLa and BHK‐21 cells (Figure 3g, Figure S4G,H), suggesting that the EVs formation contributed to viral assembly during JEV infection. On the other hand, the extracellular PFU/intracellular PFU ratios were also calculated to estimate the efficiency of viral release. Interestingly, our study revealed that GW4869 treatment inhibited viral release in PIEC and BHK‐21 cells (Figure 3h and Figure S4J). However, it demonstrated a promotive effect in HeLa cells (Figure S4I). These results suggest that the effect of EVs on viral release varies in different cell types.

### 3.4 EVs favour JEV in evading the effect of host neutralizing antibodies

**FIGURE 4:**
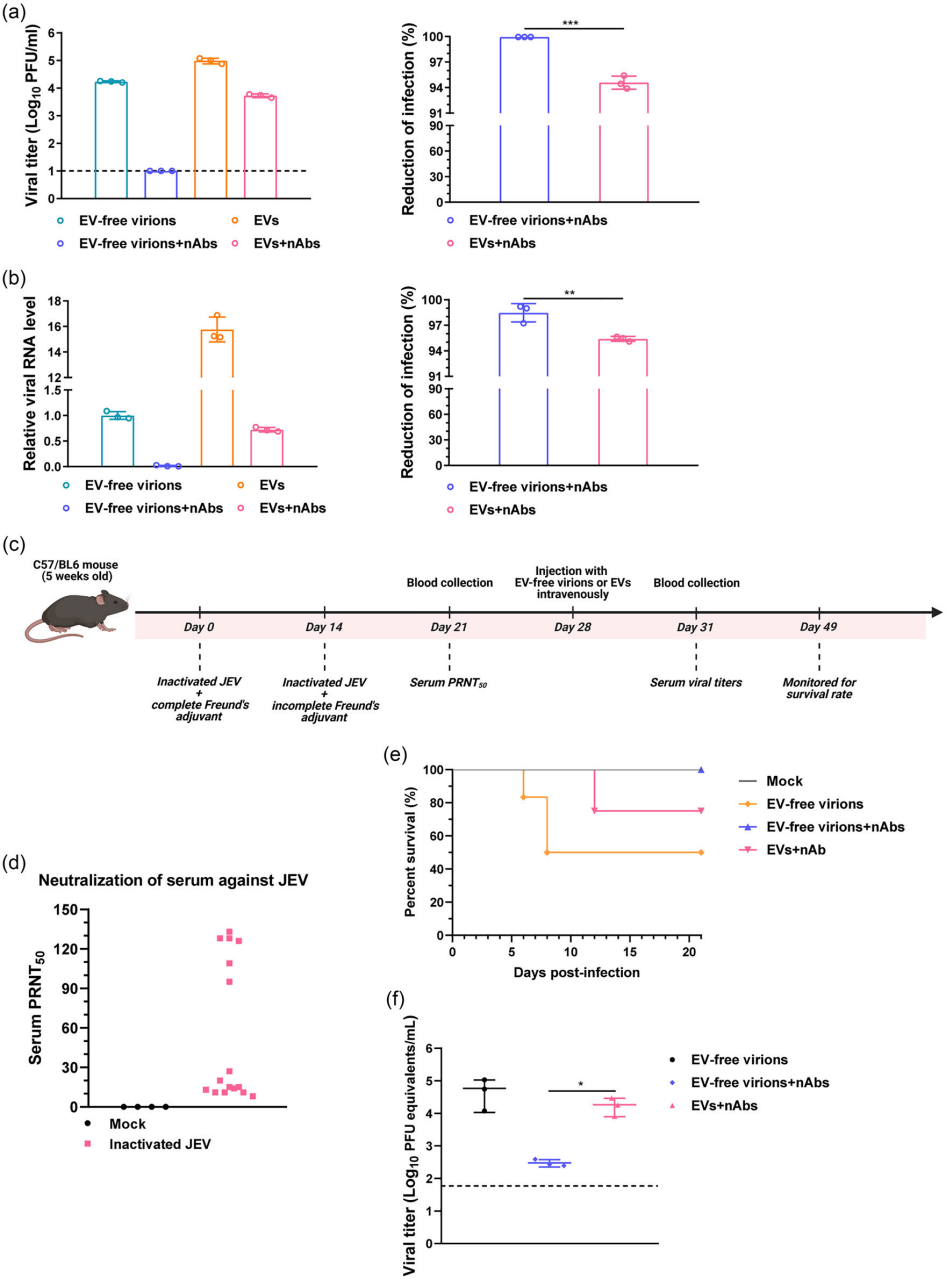
The ability of EVs to escape from neutralizing antibodies. (a)–(b) EVs or EV‐free virions were purified from BHK‐21 cells. EVs or EV‐free virions with equal copy number of viral RNA were incubated with diluted JEV nAbs for 1 h at 37°C, and the mixtures were added to BHK‐21 cells for another 1 h at 37°C. The supernatants and cells were harvested, and the viral titers in the supernatants and the intracellular viral RNA levels were quantified by plaque assay (a) and qRT‐PCR (b) at 24 hpi, respectively. Reduction of infection is defined as EV‐free virions or EVs minus EV‐free virions + nAbs or EVs + nAbs and divided by EV‐free virions or EVs, respectively. Statistical analysis, two‐tailed unpaired Student's *t*‐test. (c)–(f) Schematic diagram of mice immunization procedure and challenge regimen (c). Five‐week‐old mice were injected intramuscularly with the inactivated JEV or Mock‐EVs, respectively. The serums were collected at 7 days post the booster immunization and the neutralization antibody titers were determined by plaque reduction neutralization test (Mock, *n* = 4 per group; Inactivated JEV, *n* = 16 per group) (d). Mice were injected intravenously with BHK‐21 cell‐derived EVs and EV‐free virions containing equal viral RNA copy numbers, respectively. Survival rates were monitored for 3 weeks (*n* = 4 per group) (e). At 3 dpi, the blood was collected from mice, and the viral loads in the blood were measured by Taqman qRT‐PCR (*n* = 3 per group) (f). Data are presented as mean ± SD for in vitro experiments and as mean ± SEM for in vivo experiments. Statistical analyses were performed using two‐tailed unpaired Student's *t*‐test (a), (b), or log‐rank test (e), or one‐way ANOVA test with Dunnett post‐hoc test (f). The dotted lines represent the limit of detection. BHK‐21, baby hamster kidney; dpi, days postinfection; EVs, extracellular vesicles; hpi, hours postinfection; JEV, Japanese encephalitis virus; qRT‐PCR, quantitative reverse transcription‐PCR; SEM, standard error of mean; TEM, transmission electron microscope. ^*^ *p* < 0.05; ^**^ *p* < 0.01; ^***^ *p* < 0.001.

The virus packaged by EVs has been shown to evade the recognition of host nAbs (Morris‐Love et al., 2019; Su et al., 2021; Wang et al., 2018; Xu et al., 2020a, 2020b; Zhang et al., 2019). Therefore, a neutralization assay was conducted to examine the impact of EVs on the effect of JEV‐specific nAbs. EVs and EV‐free virions from JEV‐infected BHK‐21 cells were individually incubated with nAbs for 1 h at 37°C, and then the mixture was added to BHK‐21 cells. After incubation at 37°C for another 1 h, the unbound EVs and EV‐free virions were removed by washing with PBS, and the cells were incubated with fresh medium. The supernatants and cells were harvested at 24 hpi, and the viral titers in the supernatants and the intracellular viral RNA abundance were measured by plaque assay and qRT‐PCR, respectively. As expected, incubation with JEV nAbs blocked the viral infection in BHK‐21 cells (Figure 4a,b). However, the EVs exhibited a weak capability to counteract the neutralizing effects of nAbs (Figure 4a,b right panels). This was demonstrated by slightly more reduction of infectious virus (Figure 4a, right panel) and viral RNA (Figure 4b, right panel) produced in the cells infected with EV‐free virions than EVs, after incubation with nAbs.

Furthermore, to further validate the effect of EVs on evading the neutralizing effect of nAbs, an in vivo neutralization assay was performed using a mouse model. The immunization procedure and challenge regimen are shown (Figure 4c). Five‐week‐old mice were immunized with inactivated JEV. Following a booster immunization, the serum samples were collected, and the neutralizing antibody titers were determined (Figure 4d). Subsequently, the mice were i.v. with BHK‐21 cell‐derived EVs and EV‐free virions, both containing equal viral RNA copy numbers, respectively. The mice were monitored for survival rates, and viral loads in the blood were assessed. The results showed that, compared to the EV‐free virion‐infected mice, a slightly higher mortality rate was observed in the EV‐infected mice (Figure 4e), along with elevated viral loads in the blood (Figure 4f). These findings indicate that EVs exhibit a weak capacity to resist the neutralizing effect of nAbs both in vitro and in vivo.

### 3.5 EVs promotes JEV to cross the blood‐brain barrier and the placental barrier

**FIGURE 5:**
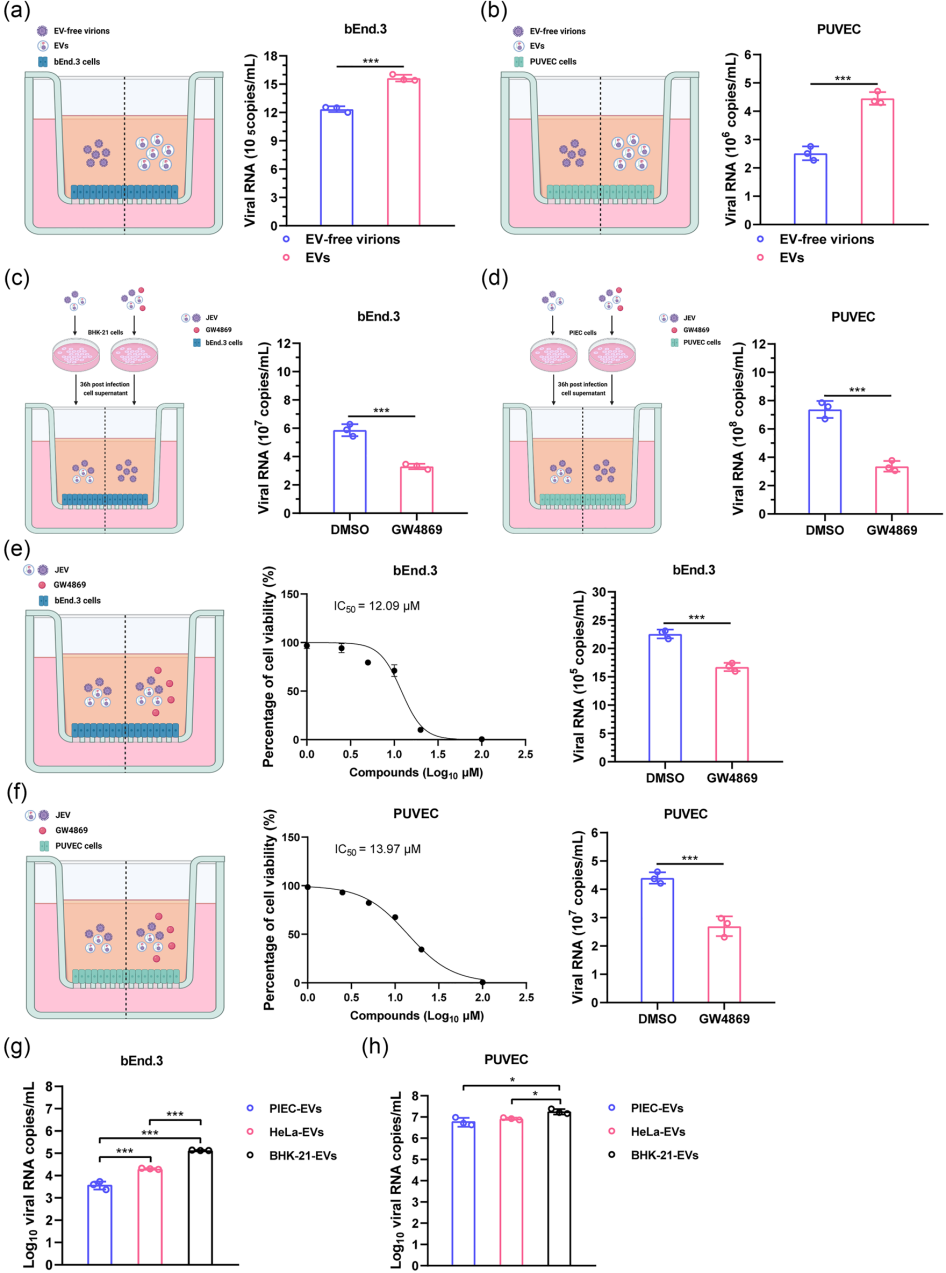
The ability of EVs to cross the blood‐brain and placental barriers. (a)–(b) The bEnd.3 (a) or PUVEC (b) cells in the upper chamber were infected with BHK‐21 or PIEC cell‐derived EVs and EV‐free virions containing equal viral RNA copy numbers, respectively. The supernatants in the lower chamber were harvested at 24 hpi, and the viral RNA levels were quantified by Taqman qRT‐PCR. (c)–(d) BHK‐21 or PIEC cells were infected with JEV, and the supernatants were removed and replaced by fresh DMEM containing DMSO or GW4869 at 1 hpi. At 36 hpi, the supernatants were harvested, and the viral RNA levels were quantified by Taqman qRT‐PCR. Followingly, the upper chamber bEnd.3 (c) or PUVEC (d) cells in the upper chamber were incubated with the supernatant from BHK‐21 or PIEC cells containing equal viral RNA copy numbers. Finally, the supernatants in the lower chamber were harvested at 24 hpi, and the viral RNA levels were quantified by Taqman qRT‐PCR. (e)–(f) bEnd.3 (e) or PUVEC (f) cells were incubated with different concentrations of GW4869, and cell viability was measured and analysed (middle panel). The bEnd.3 or PUVEC cells in the upper chamber were infected with JEV, followed by treating with DMSO or GW4869 at 1 hpi. The supernatants in the lower chamber were harvested at 24 hpi, and the viral RNA levels were quantified by Taqman qRT‐PCR. (g)–(h) The bEnd.3 (g) or PUVEC (h) cells in the upper chamber were inoculated with PIEC, HeLa and BHK‐21 cell‐derived EVs containing equal viral RNA copy numbers, and the supernatants in the lower chamber were harvested at 24 hpi, and the viral RNA levels were quantified by Taqman qRT‐PCR. Data are presented as mean ± SD from three independent experiments in duplicate. Statistical analyses were performed using two‐tailed unpaired Student's *t*‐test (a)–(f), or one‐way ANOVA test with Dunnett post‐hoc test (g) and (h). BHK‐21, baby hamster kidney cells; EVs, extracellular vesicles; HeLa, Henrietta Lacks; hpi, hours postinfection; JEV, Japanese encephalitis virus; PIEC, porcine iliac artery endothelial cells; PUVEC, porcine umbilicus vein endothelial cells; qRT‐PCR, quantitative reverse transcription‐PCR. ^*^ *p* < 0.05; ^***^ *p* < 0.001.

Crossing the tissue barriers is a crucial step for JEV to cause neurological diseases in humans and reproductive diseases in pigs. To understand the role of EVs in regulating the ability of JEV to cross the tissue barriers, the in vitro models of blood‐brain barrier and placental barrier were constructed by growing bEnd.3 or PUVEC cells on a transwell system, respectively. The cells in the upper chamber were inoculated with BHK‐21 or PIEC cell‐derived EVs and EV‐free virions containing equal viral RNA copy numbers, and the supernatants in the lower chamber were harvested and quantified by Taqman qRT‐PCR. Our results showed that inoculation with EVs rather than EV‐free virions resulted in a significantly higher abundance of viral RNA in the lower chamber of both bEnd.3 and PUVEC transwell systems, with increases of 22.90% and 71.66%, respectively (Figure 5a,b), indicating that EVs are more efficient in crossing both the blood‐brain barrier and the placental barrier. To further confirm this finding, BHK‐21 and PIEC cells were infected with JEV at 1 MOI followed by treatment of GW4869 or DMSO, and the supernatants were collected and quantified at 36 hpi. The upper chamber of bEnd.3 or PUVEC cells were then inoculated with the supernatant containing equal viral RNA copy numbers of JEV from BHK‐21 or PIEC cells, respectively. Then viral RNA in the lower chamber were quantified by Taqman qRT‐PCR. The results indicated a decrease in the RNA levels of JEV in the transwell systems inoculated with the supernatant from GW4869‐treated cells, showing reductions of 46.59% and 51.18%, respectively, when compared to untreated cells (Figure 5c,d). These results supported that EVs were critical for JEV to cross the endothelial barriers. To investigate if EVs released by endothelial cells could also contribute to assisting JEV in crossing the tissue barriers, bEnd.3 and PUVEC cells were infected with JEV followed by treatment with GW4869, and viral RNA in the lower chamber were quantified by Taqman qRT‐PCR. We found that GW4869 treatment on bEnd.3 cells and PUVEC resulted in significant reductions in viral RNA levels, with decreases of 22.92% and 41.44%, respectively (Figure 5e,f), suggesting that EVs produced by endothelial cells play a crucial role in viral transmission across the blood‐brain barrier and the placental barrier.

To further investigate the specificity of EVs from different origins that are permitted to enter the brain through the blood‐brain barrier and the placental barrier, EVs derived from various cell types infected with JEV, with equal viral RNA copy numbers, were incubated with bEnd.3 and PUVEC cell‐based barrier models. The efficiency of EVs from different cell types in traversing the barrier models was assessed by quantifying the viral RNA copies present in the lower chamber medium. The results demonstrated a variation in the efficiency of various cell‐derived EVs in crossing tissue barriers. Notably, EVs derived from BHK‐21 cells displayed the highest efficacy in crossing both the in vitro blood‐brain barrier and placental barrier models (Figure 5g,h). This finding suggests a specificity of EVs based on their origin in penetrating tissue barriers.

### 3.6 EVs enhances the pathogenicity of JEV in vivo

**FIGURE 6:**
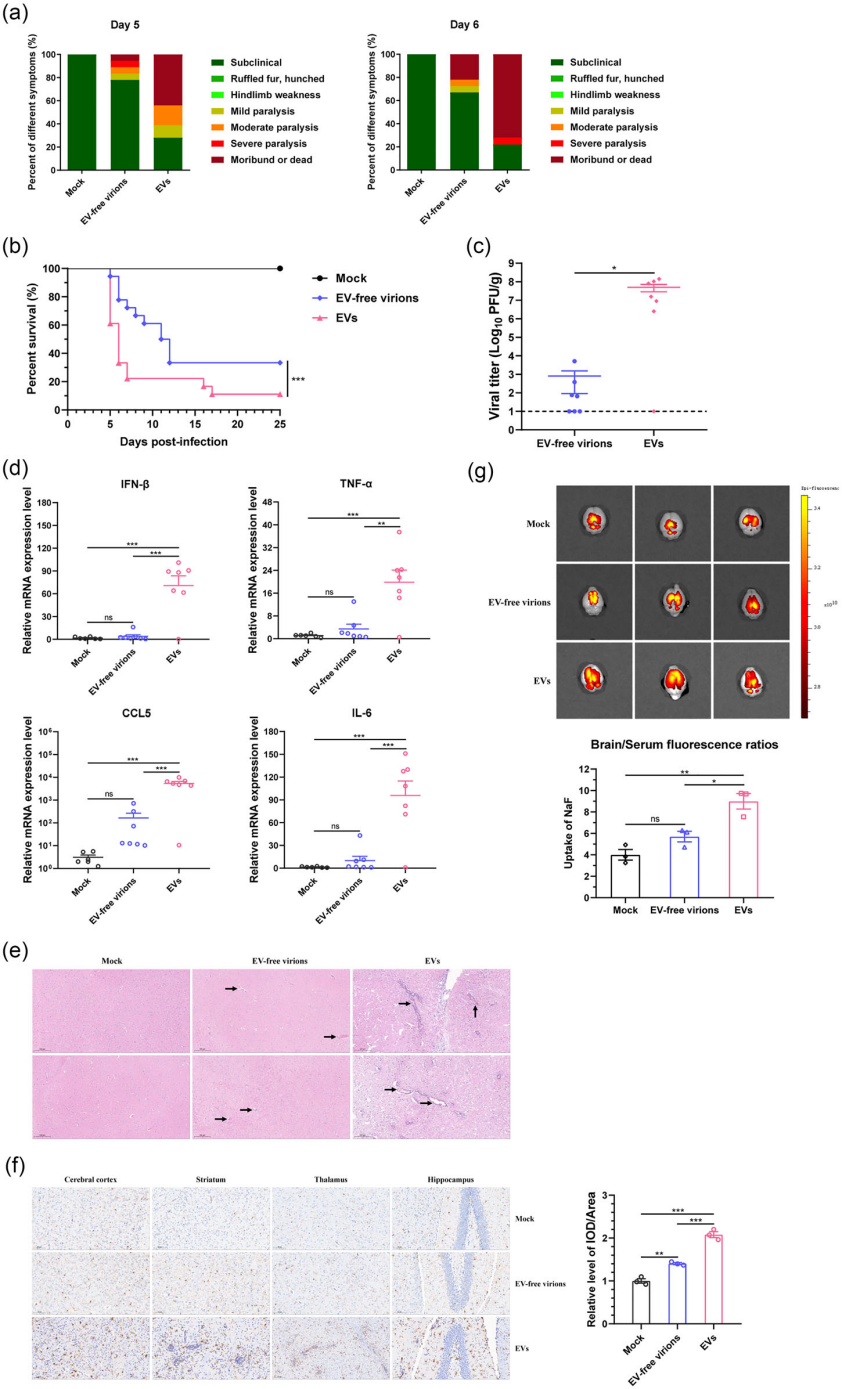
The effect of EVs on JEV infection in mice. Five‐week‐old C57/BL6 mice were injected with EVs and EV‐free virions derived from BHK‐21 cells containing equal viral RNA copy numbers via tail vein, respectively. The mice injected with uninfected cell‐derived EVs were considered as a mock group. Clinical symptoms (a) and survival rates (b) were monitored (*n* = 18 mice per group). At 5 dpi, mice from each group were sacrificed, and the viral loads (c) and the mRNA levels of IFN‐β, TNF‐α, CCL5, and IL‐6 (d) in the brain tissues were measured by plaque assay and qRT‐PCR, respectively (*n* = 7 mice per group). The pathological changes in the brain tissues were examined by H&E staining, with the vascular cuff indicated by black arrows (*n* = 3 mice per group) (e). IHC staining of brains was performed to determine the expression of IBA‐1 protein, and the IOD analysis was calculated from three mice with four visual fields per mouse using Image‐Pro Plus (*n* = 3 mice per group) (f). To assess the permeability of BBB, NaF was injected into 10 mg. The content of NaF in brain tissue was measured by using Spark10M Spectrophotometers (g, upper panel). The NaF uptake efficiency was determined by the ratio of fluorescence intensity in brain tissue / brain tissue weight to fluorescence intensity in serum / blood volume (g, lower panel) (*n* = 3 mice per group). Data are pooled from two independent experiments. The representative images are shown in (e), (f) and (g). Data are presented as mean ± SEM. Statistical analyses were performed using log‐rank test (b), or one‐way ANOVA test with Dunnett post‐hoc test (c), (d), (f) and (g). The dotted line represents the limit of detection. Scale bar: 200 µm (e), 100 µm (f). BHK‐21, baby hamster kidney cells; dpi, days postinfection; EVs, extracellular vesicles; H&E, hematoxylin and eosin; IHC, immunohistochemistry; IOD, integrated option density; JEV, Japanese encephalitis virus; qRT‐PCR, quantitative reverse transcription‐PCR; SEM, standard error of mean. ^*^ *p* < 0.05; ^**^ *p* < 0.01; ^***^ *p* < 0.001.

To evaluate the in vivo function of EVs, mice were i.v. with EVs and EV‐free virions, containing equal viral RNA copy numbers, respectively. The mice were then observed daily for 4 weeks to monitor clinical symptoms and survival rates. The results showed that compared with EV‐free virion‐infected mice, the EV‐infected mice showed more severe symptoms (Figure 6a) and a significant reduction in survival rates (Figure 6b). Additionally, viral loads and the expression of inflammatory cytokines in the brain tissues of mice from each group were measured at 5 dpi. The results showed higher levels of viral loads and inflammatory cytokines including IFN‐β, TNF‐α, CCL5, and IL‐6 in brains of EV‐infected mice, as compared to those in EV‐free virion‐infected mice (Figure 6c,d). To further assess the pathological changes in brain tissue, brain samples collected at 8 dpi were subjected to H&E and IHC staining. The brains of mice infected with EVs displayed perivascular cuffing; however, these indicators of encephalitis were alleviated in mice infected with EV‐free virions (Figure 6e). IHC staining was performed by using the IBA‐1 antibody to identify microglia. It was observed that there were more activated microglia in the brain tissues of EV‐infected mice compared to that of EV‐free virion‐infected mice (Figure 6f). Moreover, to investigate whether the distinct pathogenicity between EVs and EVs‐free virions was associated with differences in BBB permeability contributing to brain infection, the NaF was i.p. into the infected mice. At 5 dpi, the fluorescent intensity in the brain tissue of mice were observed and quantified using a small animal IVIS. The results showed that, compared to EV‐free virion‐infected mice, those infected with EVs exhibited a more pronounced influx of NaF into the brain, indicating more severe damage of the BBB (Figure 6g). These results demonstrated that EVs promoted the infection of JEV in mouse model, resulting in more severe symptoms.

**FIGURE 7:**
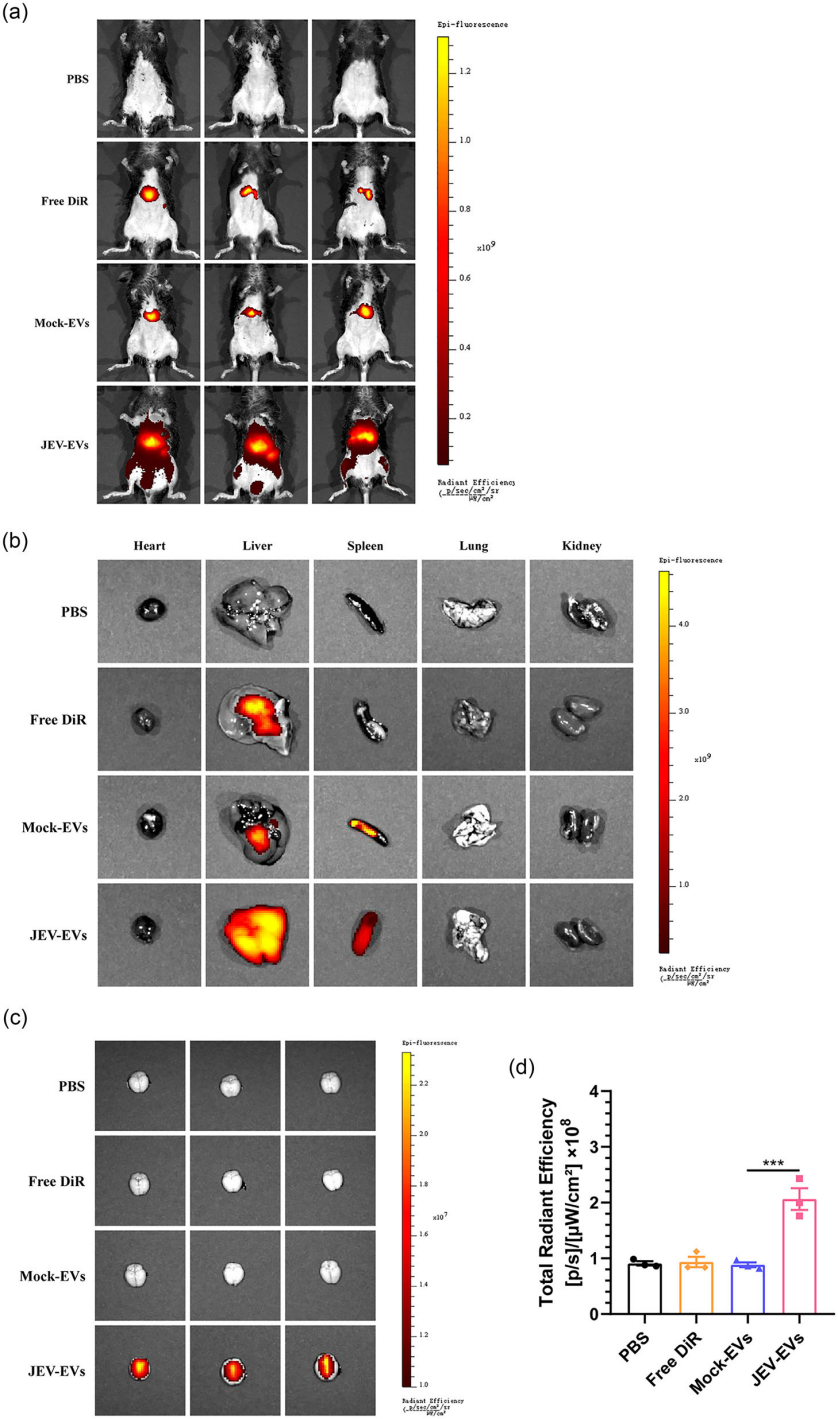
The biodistribution of EVs in mice. EVs derived from JEV or mock‐infected BHK‐21 cells were labelled with DiR (Mock‐EVs and JEV‐EVs). These DiR‐labelled EVs were then injected into the mice via tail vein. Mice injected with PBS or free DiR were considered as control. At 4 hpi, the biodistribution of DiR‐labelled EVs in the mouse bodies (a), various peripheral organs (b), and brains (c) was evaluated by an in vivo optical imaging system (IVIS spectrum, Perkin Elmer), respectively (*n* = 3 mice per group). The fluorescence intensity in brains were shown as mean ± SEM from three mice. The statistical analysis was performed using one‐way ANOVA test with Dunnett post‐hoc test. ^***^p < 0.01. hpi, hours postinfection; EVs, extracellular vesicles; JEV, Japanese encephalitis virus; SEM, standard error of mean.

Additionally, to further visualize the biodistribution of EVs in vivo, the DiR‐labelled EVs were injected into the mice via the tail vein. The biodistribution of these DiR‐labelled EVs in the mouse was evaluated by an IVIS. The results showed a representative organ distribution of EVs in the mouse model. After 4 h postinjection, the EVs were found to have disseminated to the liver and spleen (Figure 7a). Consistently, tissue dissection and imaging analysis also showed that the EVs were predominantly detected in the liver and spleen (Figure 7b). Moreover, we observed that mice injected with EVs derived from JEV‐infected cells exhibited a higher fluorescence intensity in the brain compared to those receiving EVs from mock‐infected cells (Figure 7c,d), suggesting an enhanced capability of EVs to cross tissue barriers following JEV infection.

To further assess the effects of EVs on JEV infection in vivo, C57BL/6 mice were i.p. with BHK‐21 cell‐derived EVs and EV‐free virions, containing equal viral RNA copy numbers, respectively. Consistently, the resulted showed that compared with EV‐free virion‐infected mice, the EV‐infected mice showed more severe symptoms (Figure S5A,B) and higher mortality rate (Figure S5C). Furthermore, elevated levels of viral loads and inflammatory cytokines were detected in the brains of EV‐infected mice, as compared to those infected with EV‐free virions (Figure S5D,E). Additionally, the brains of EV‐infected mice displayed more perivascular cuffing and activated microglia, but these indicators of encephalitis were alleviated in EV‐free virion‐infected mice (Figure S5F,G). To determine whether the differences in JEV infection between EVs and EV‐free virions in mice were species‐dependent, similar in vivo experiments were conducted using PIEC‐derived EVs and EV‐free virions. Similarly, infection with EVs led to more severe symptoms (Figure S6A,B) and a higher mortality rate (Figure S6C). These findings indicate that EVs originating from PIEC cells also enhance JEV infection in vivo. The biodistribution of DiR‐labelled EVs in mice after intraperitoneal injection was also performed. The results showed that the EVs were observed to disseminate to all vital organs at 12 h postinjection, with the majority of the DiR‐labelled EVs were primarily localized in the liver and intestinal tract (Figure S7A). Tissue dissection and imaging analysis further confirmed that the EVs were predominantly detected in the liver, kidney and intestinal tract (Figure S7B). These results demonstrated that EVs promoted the infection of JEV in mouse model, resulting in enhanced pathogenicity.

## 4 DISCUSSION

**FIGURE 8:**
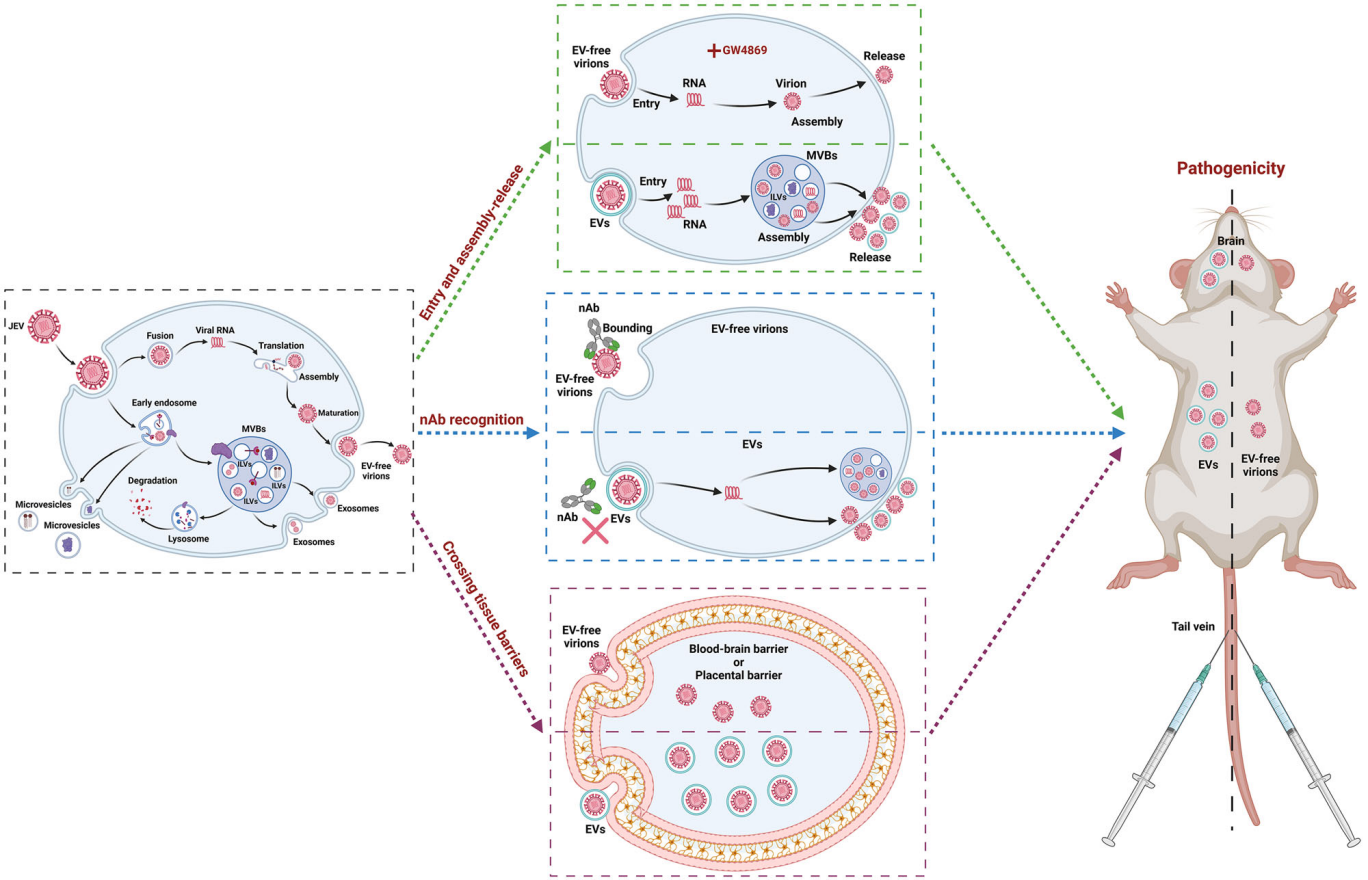
Proposed model of JEV hijacking the EVs to promote viral infection and pathogenicity. During JEV infection, virions are released in two different pathways: EV‐free virions and virion‐containing EVs. In the case of infection with EV‐free virions, the virions initially bind to cellular receptors and enter host cells through endocytosis. In the case of infection with virion‐containing EVs, these vesicles mediate viral invasion via a receptor‐binding independent way. During viral assembly and release process, MVBs selectively package the viral genome, viral proteins, and virions before fusing with the cell membrane to be released into extracellular space, forming EVs. Inhibition of EV generation by treating with GW4869 would suppress this process, thereby inhibiting viral assembly‐release. In addition, EV‐free virions undergo modifications by organelles such as endoplasmic reticulum and Golgi apparatus before being released into extracellular space. Furthermore, EVs contribute to viral escape from neutralizing antibodies and crossing the blood‐brain and placental barriers, thereby enhancing viral infection and pathogenicity in vivo. Schematic diagram was created using BioRender (BioRender.com). EVs, extracellular vesicles; JEV, Japanese encephalitis virus; MVBs, multivesicular bodies.

Japanese encephalitis is an important vector‐borne zoonotic disease that continues to be a concern due to its potential global publish health threat. It is characterized by distinct clinical features, including neurological sequelae in human, abortions in sows, and orchitis in boars (Campbell et al., 2011; Cappelle et al., 2016; Fulmali et al., 2011; Kulkarni et al., 2018; Lim et al., 2022; Tarantola et al., 2014; van den Hurk et al., 2003; Zheng et al., 2019). However, effective therapies for JEV infection are still lacking. As a secretion pathway, EVs have been found to play an important role in viral infection (Chen et al., 2021; Cortes‐Galvez et al., 2023; Morris‐Love et al., 2019; Wang et al., 2019, 2018). In this study, we demonstrated that EVs released by JEV‐infected cells contain JEV virions and exhibit the ability to infect host cells. Further investigation revealed the significant roles of EVs in various processes of JEV infection, including viral replication, evading nAbs, and crossing tissue barriers, which may contribute to the infectivity and pathogenicity of JEV (Figure 8). These findings provide us novel understandings for the mechanism of JEV infection and pathogenesis.

Currently, the available methods for EV isolation mainly include UC, density DG, polymer precipitation (PP), size exclusion chromatography (SEC), ultrafiltration (UF) and IC (Arab et al., 2019; McNamara et al., 2019; Onódi et al., 2018; Reyes‐Ruiz et al., 2020; Rider et al., 2016). Our study found that the isolation and purification of EVs from JEV‐infected cells can be effectively achieved using a combination of UC and density gradient centrifugation, due to its universality, applicability, no need for antibodies, suitable for large volume, high purity, and low cost. Our results showed that UC‐DG method could be used to isolate and purify EVs from cells of different species, with EVs in F7 and EV‐free virions in F10, which is consistent with previous reports about human cytomegalovirus (HCMV) (Streck et al., 2020), encephalomyocarditis virus (EMCV) (van der Grein et al., 2019), hepatitis A virus (HAV) (Feng et al., 2013), and Langat virus (LGTV) (Zhou et al., 2018).

Importantly, our study revealed that EVs derived from JEV‐infected cells enclose intact virions, as observed through immunoelectron microscope. Similar findings have been reported in EVs derived from cells infected with dengue virus (DENV) (Reyes‐Ruiz et al., 2019), a flavivirus close related to JEV, as well as other viruses, such as rice dwarf virus (RDV) (Chen et al., 2021), CSFV (Wang et al., 2022), HAV (Feng et al., 2013), and hepatitis B virus (HBV) (Wu et al., 2023). However, this phenomenon has not been observed in EVs from cells infected with another flavivirus, ZIKV (Huang et al., 2018; Martínez‐Rojas et al., 2020; York et al., 2021; Zhao et al., 2023). Additionally, the enrichment of viral proteins, including the M and E proteins, in F7, which exhibited high infectivity to host cells, further supports the presence of JEV virions within EVs. Moreover, our findings showed that EVs could be released through the fusion of virion‐containing MVB with cell membrane. This observation greatly enhanced our understanding of the mechanisms of EV formation.

Our study demonstrated that EVs enhanced JEV multiplication in PIEC and BHK‐21 cells. Additionally, our further investigation suggested that EVs may play a crucial role in promoting both viral entry and assembly‐release processes during JEV life cycle. In the early stage of viral infection, virion‐containing EVs can directly fuse with cell membrane, releasing the viral contents into cells, while EV‐free virions invade cells through receptor binding and endocytosis. During the late stage of viral replication, MVB selectively packages the partial or full‐length viral genome, viral proteins, immature or mature virions and fuses with cell membrane to be released into extracellular space (Chen et al., 2021; Mao et al., 2020; Wang et al., 2018), thereby enhancing viral assembly and release efficiency. However, EV‐free virions undergo modifications by organelles such as ribosomes, endoplasmic reticulum and Golgi apparatus before they can be released into extracellular space. These findings provided explanations for the effect of EVs on regulating viral entry, assembly and release.

However, our results showed that after treatment with EVs inhibitor GW4869, JEV multiplication was enhanced in HeLa cells, which was opposite to that in PIEC and BHK‐21 cells, suggesting the role of EVs in JEV propagation differs across species. While HeLa‐derived EVs were found to contain viral proteins and virions, they were unable to facilitate viral infection. This could be attributed to the presence of anti‐viral components within EVs, such as proteins, mRNAs, and microRNAs originating from the host. For instance, EVs released by HSV‐1‐infected cells have been reported to carry innate immune components (such as STING) and other host and viral factors, which can activate innate immune responses in recipient cells to inhibit HSV‐1 replication (Deschamps & Kalamvoki, 2018). Another study has demonstrated that EVs extracted from FMDV‐infected PK‐15 cells pack with miRNA‐136, in turn inhibiting viral proliferation (Xu et al., 2020b). Moreover, RV infection has been found to up‐regulate miR‐423‐5p expression in EVs, resulting in feedback inhibition of RV replication by abrogating the inhibitory effect of suppressor of cytokine signalling 3 (SOCS3) on type I interferon signalling (Wang et al., 2019). Further studies will be conducted to better elucidate the mechanism of functional differentiation of EVs in different species. However, confirmation of this speculation necessitates additional verification through multi‐omics sequencing analysis.

Many studies have reported that EVs from virus‐infected cells have the ability to evade nAbs, such as JCPyV (Morris‐Love et al., 2019), FMDV (Xu et al., 2020b; Zhang et al., 2019), SVV (Xu et al., 2020a), REV (Su et al., 2021), PRRSV (Wang et al., 2018), and CSFV (Wang et al., 2022). Consistently, our study revealed that EVs derived from JEV‐infected cells also possess the ability to evade nAbs both in vitro and in vivo, although this resistant effect is relatively weak. This phenomenon may occur due to the capability of nAbs to bind to EV‐free virions, hindering their attachment to cells and consequently preventing infection. However, it is important to note that nAbs is unable to recognize EVs. As a result, EVs can release viral components into the cytoplasm through membrane fusion.

Notably, our study demonstrated that compared to EV‐free virions, EVs from JEV‐infected cells were more effectively in assisting the virus to cross the blood‐brain barrier and the placental barrier. This may be due to the potential role of EVs in facilitating JEV infection on endothelial cells and enhancing viral release from these cells. Additionally, EVs may also mediate the ability to cross the endothelial barrier via the intercellular pathway. These functions of EVs may contribute to the development of diseases caused by JEV infection, such as encephalitis in human, orchitis in boar, and abortion in sow.

While there have been numerous studies on EV function in viral infection, it is important to note that the majority studies have been limited to in vitro experiments. Therefore, further investigation is needed to understand the role of EVs in vivo. Our study aimed to address this gap by exploring the effects of EVs purified from JEV‐infected cells in a mouse model. Our findings revealed that compared to EV‐free virions, EVs obtained from JEV‐infected cells were more efficient in establishing viral infection in a mouse model, resulting in more severe symptoms and higher mortality rate. Several previous studies have also highlighted the significance of EVs in understanding in vivo infection of viruses. For example, EVs obtained from FMDV‐infected cells are injected into C57BL/C suckling mice, resulting in productive infection and death (Zhang et al., 2019). ZIKV E proteins are found to be located on the surface of purified EVs, thus further relieving ADE‐induced exacerbation of ZIKV infection in vivo (Zhao et al., 2023). EVs purified from ALV‐J‐infected rooster seminal plasma can transmit ALV‐J infection to specific pathogen free (SPF) hens, and subsequently mediate vertical transmission of ALV‐J from hens to their progeny chicks (Liao et al., 2022). However, the cytotoxicity of EVs inhibitor GW4869 on in vivo models poses a challenge in determining the impact of EVs on the immunity and pathogenicity of virus in vivo. Future research utilizing gene‐knockout mice may be necessary.

We labelled EVs with DiR and injected them into mice either intraperitoneally or intravenously to trace the biodistribution of EVs in vivo. The results from the intraperitoneal injection showed that EVs spread to all vital organs in the mouse, while the intravenous injection of EVs resulted in a predominant distribution of EVs in the liver and spleen, with notable presence in brain tissue. These findings greatly enhance our understanding of EV transport in vivo, laying a scientific foundation for utilizing EVs as carriers for drug or small molecule delivery.

In summary, our study developed an applicable method for the isolation and purification of EVs from JEV‐infected cells of various species. These purified EVs were found to carry virions and were able to infect host cells. We observed that EVs played a crucial role in facilitating JEV propagation by promoting viral entry and assembly‐release. Additionally, we found that EVs contributed to viral escape from nAbs and crossing the blood‐brain and placental barriers. Importantly, our findings highlighted the significance of EVs in viral infection in vivo. These findings not only deepen our understanding of the roles of EVs in JEV infection, but also provide valuable clues for the development of JE therapy.

## AUTHOR CONTRIBUTIONS

**Junyao Xiong**: Conceptualization (equal); data curation (equal); formal analysis (equal); investigation (equal); methodology (equal); project administration (equal); resources (equal); software (equal); visualization (equal); writing—original draft (equal); writing—review and editing (equal). **Ling'en Yang**: Investigation (equal); software (equal); visualization (equal). **Xiaowei Nan**: Data curation (equal); formal analysis (equal). **Shuo Zhu**: Project administration (equal); visualization (equal). **Mengxue Yan**: Methodology (equal). **Shengxian Xiang**: Data curation (equal). **Luping Zhang**: Methodology (equal). **Qi Li**: Formal analysis (equal). **Chengjie Yang**: Methodology (equal). **Xugang Wang**: Methodology (equal). **Ning Wei**: Software (equal). **Huanchun Chen**: Resources (equal); supervision (equal). **Youhui Si**: Resources (equal); supervision (equal). **Shengbo Cao**: Conceptualization (equal); funding acquisition (lead); resources (equal); supervision (equal); visualization (equal); writing—original draft (equal). **Jing Ye**: Conceptualization (equal); funding acquisition (lead); resources (equal); supervision (equal); validation (equal); visualization (equal); writing—original draft (equal); writing—review and editing (equal).

## CONFLICT OF INTEREST STATEMENT

The authors declare that they have no conflict of interest.

## Supporting information



Figure S1. Conditions for EV isolation from JEV‐infected cells.

Figure S2. Purification of EVs from JEV‐infected HeLa cells via immunocapture methods.

Figure S3. Impact of EVs on JEV multiplication in HeLa and BHK‐21 cells.

Figure S4. Impact of EVs on JEV entry, assembly and release in HeLa and BHK‐21 cells.

Figure S5. The effect of EVs on JEV infection in mice via intraperitoneal injection.

Figure S6. The effect of PIEC‐derived EVs on JEV replication in mice.

Figure S7. The biodistribution of EVs i.p. in mice.

Supplementary figures.
