# Supplementary figures and images for: Extracellular vesicles promote the infection and pathogenicity of Japanese encephalitis virus
Source: J Extracell Vesicles. 2025 Jan 9;14(1):e70033. doi: 10.1002/jev2.70033 (PMC11714208; doi:10.1002/jev2.70033)

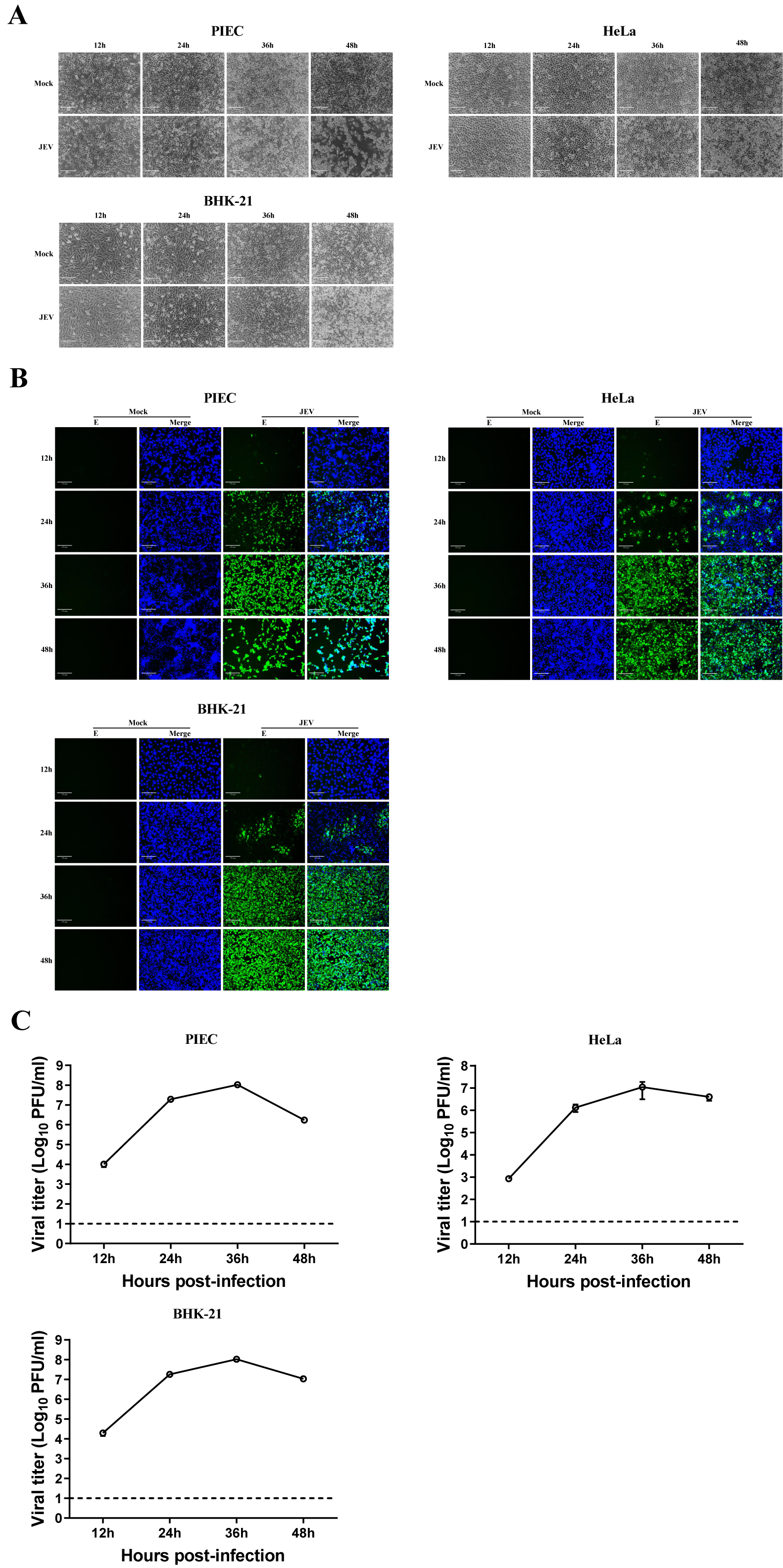

Supplement: Supplementary file 1 — Figure S1. Conditions for EV isolation from JEV‐infected cells. [file JEV2-14-e70033-s004.tif]

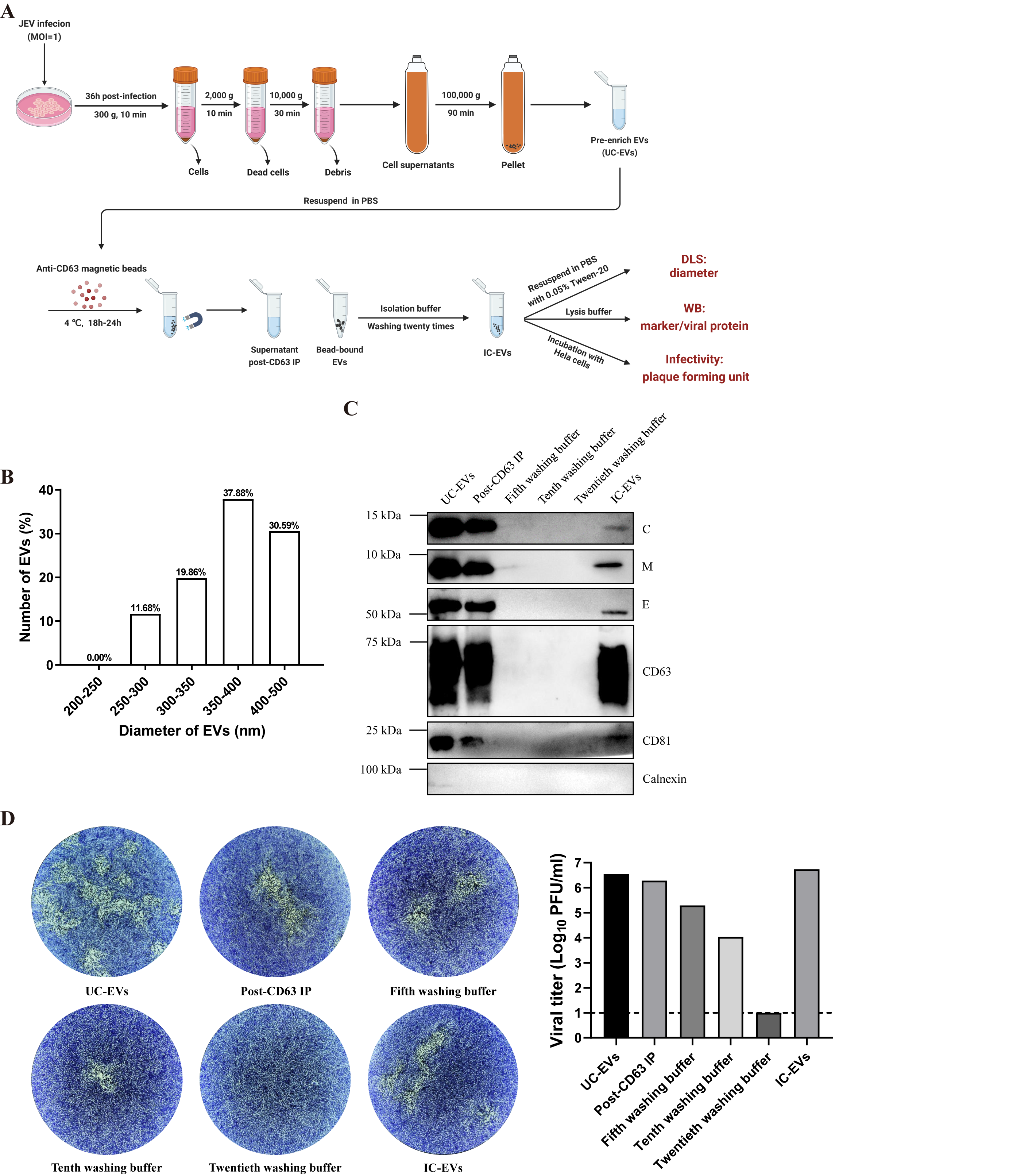

Supplement: Supplementary file 2 — Figure S2. Purification of EVs from JEV‐infected HeLa cells via immunocapture methods. [file JEV2-14-e70033-s008.tif]

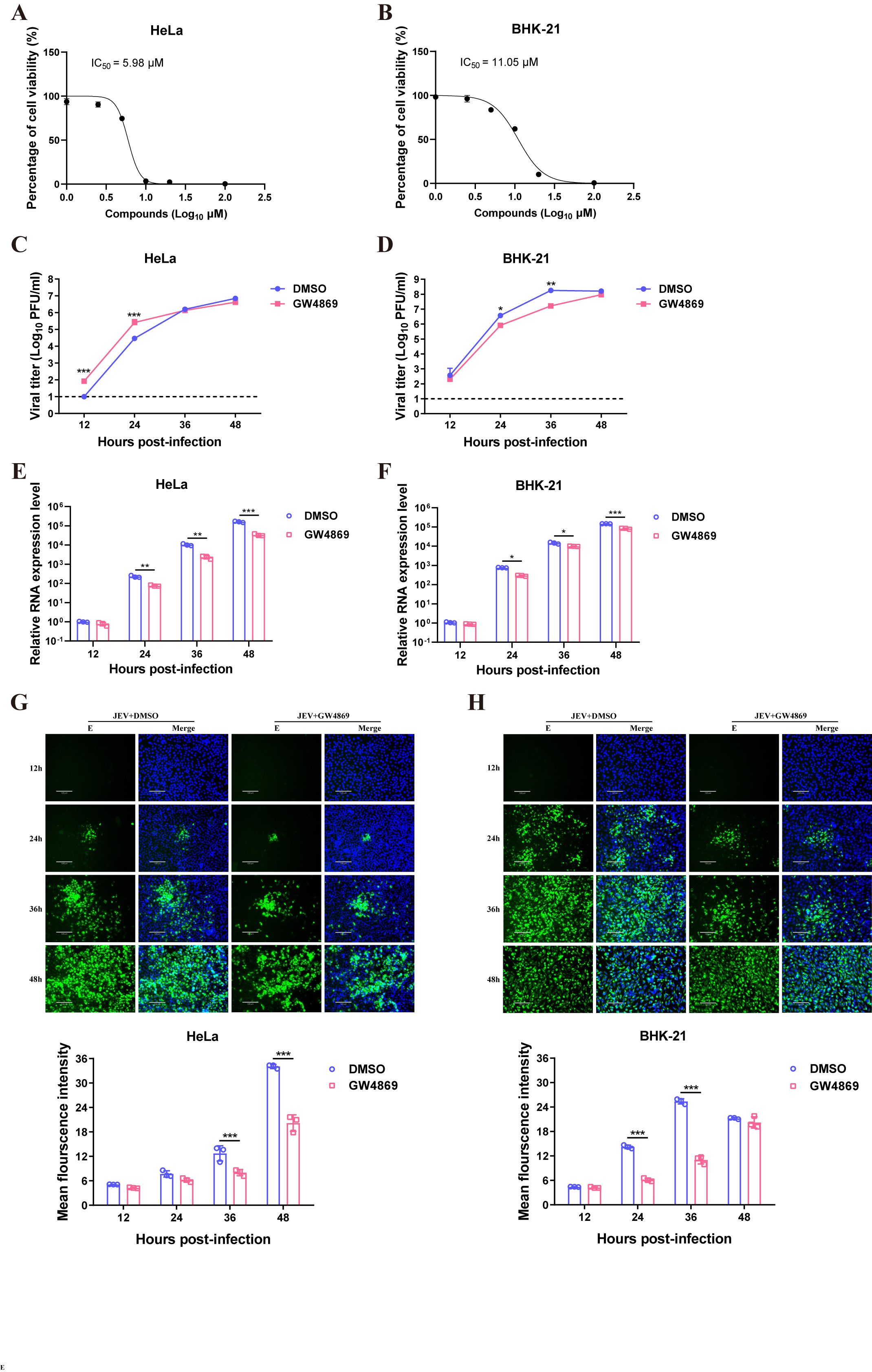

Supplement: Supplementary file 3 — Figure S3. Impact of EVs on JEV multiplication in HeLa and BHK‐21 cells. [file JEV2-14-e70033-s002.tif]

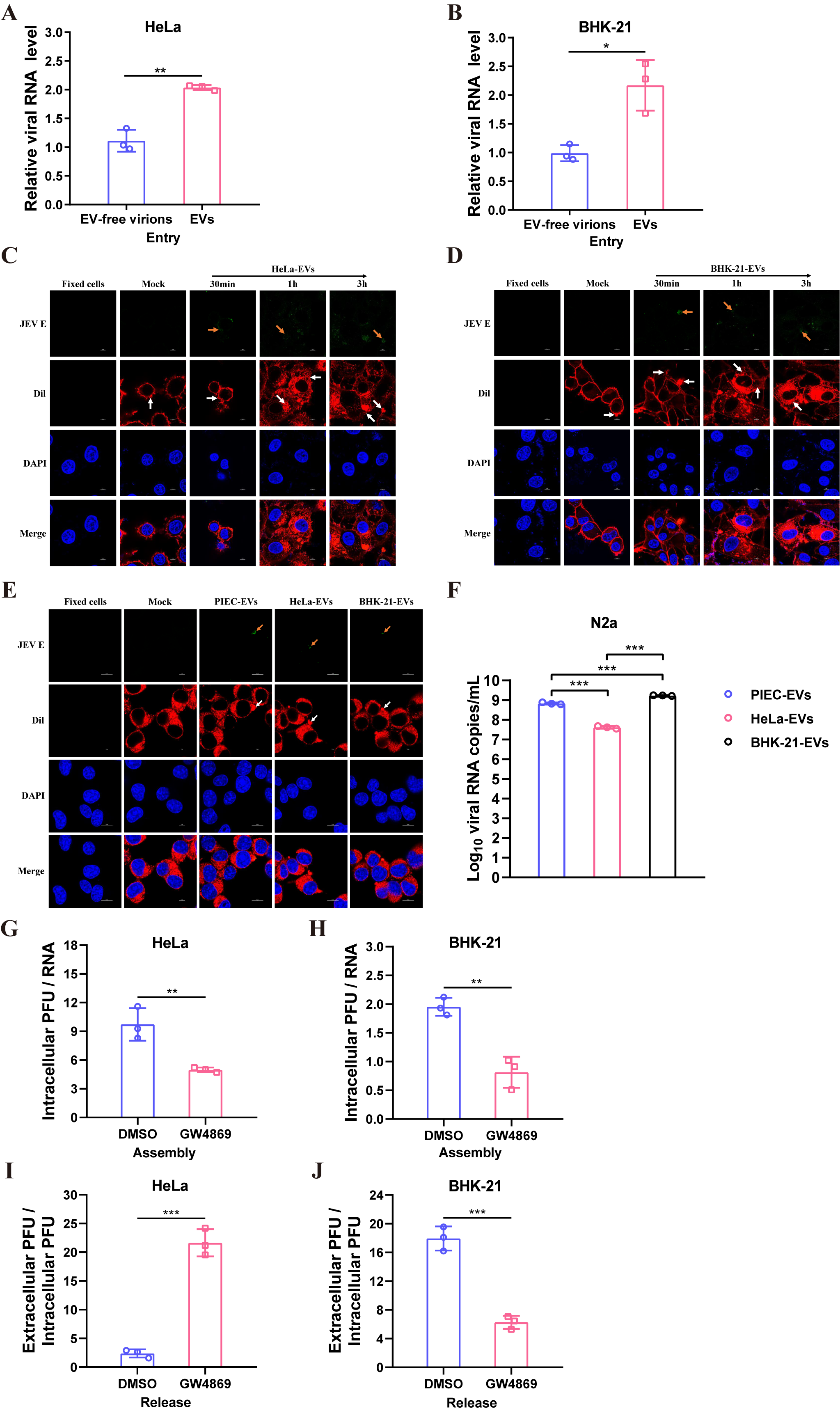

Supplement: Supplementary file 4 — Figure S4. Impact of EVs on JEV entry, assembly and release in HeLa and BHK‐21 cells. [file JEV2-14-e70033-s006.tif]

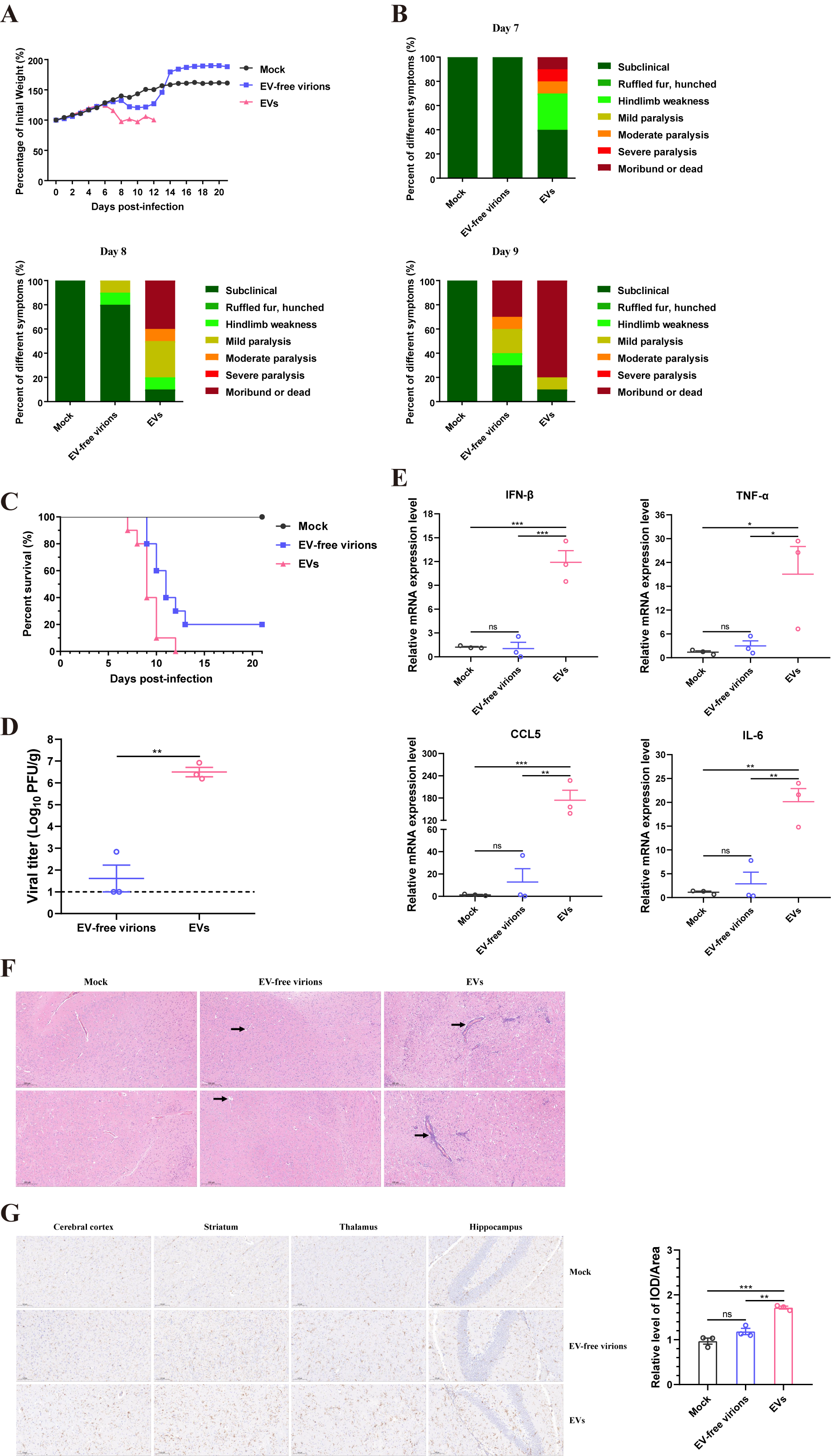

Supplement: Supplementary file 5 — Figure S5. The effect of EVs on JEV infection in mice via intraperitoneal injection. [file JEV2-14-e70033-s005.tif]

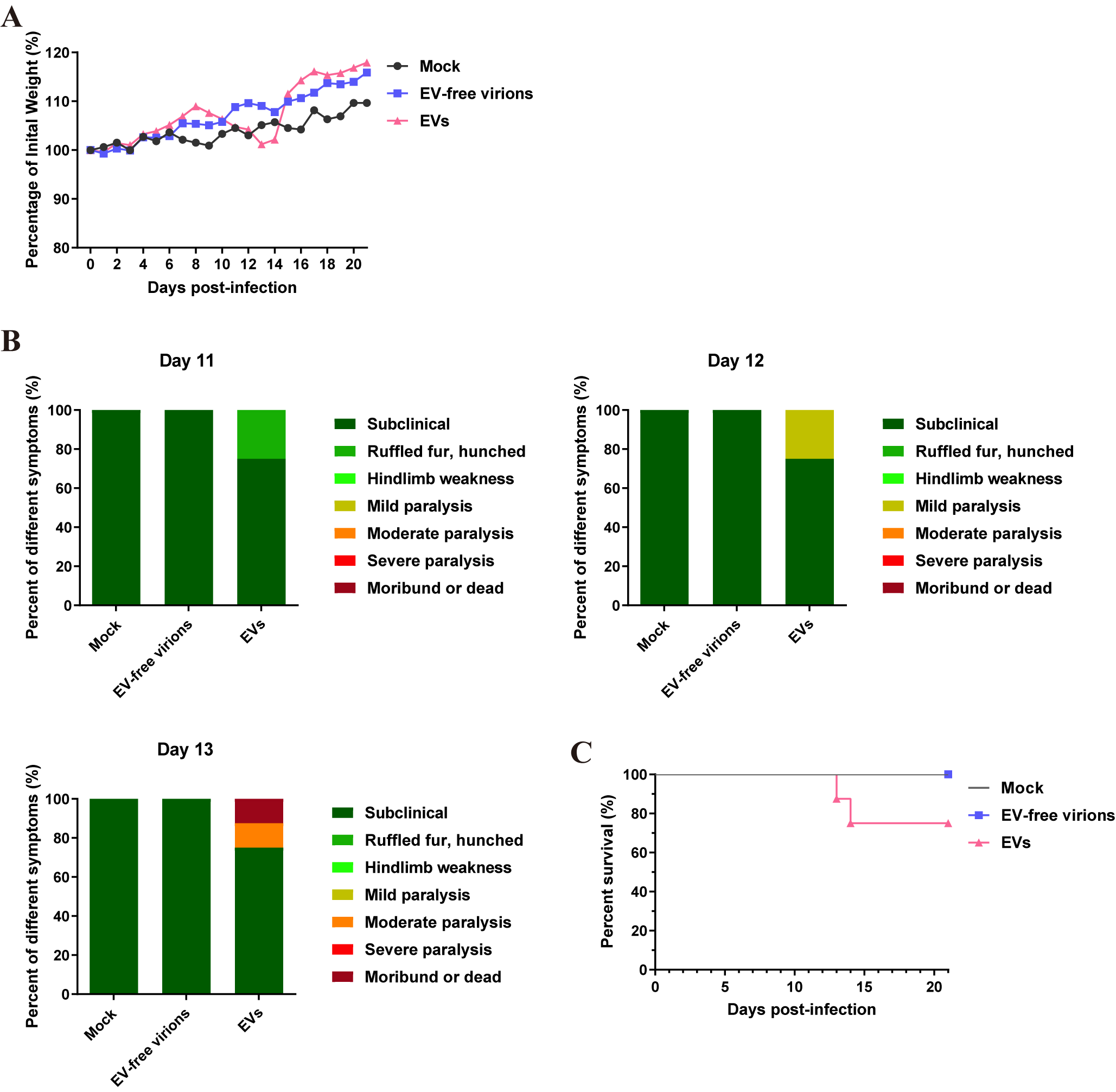

Supplement: Supplementary file 6 — Figure S6. The effect of PIEC‐derived EVs on JEV replication in mice. [file JEV2-14-e70033-s003.tif]

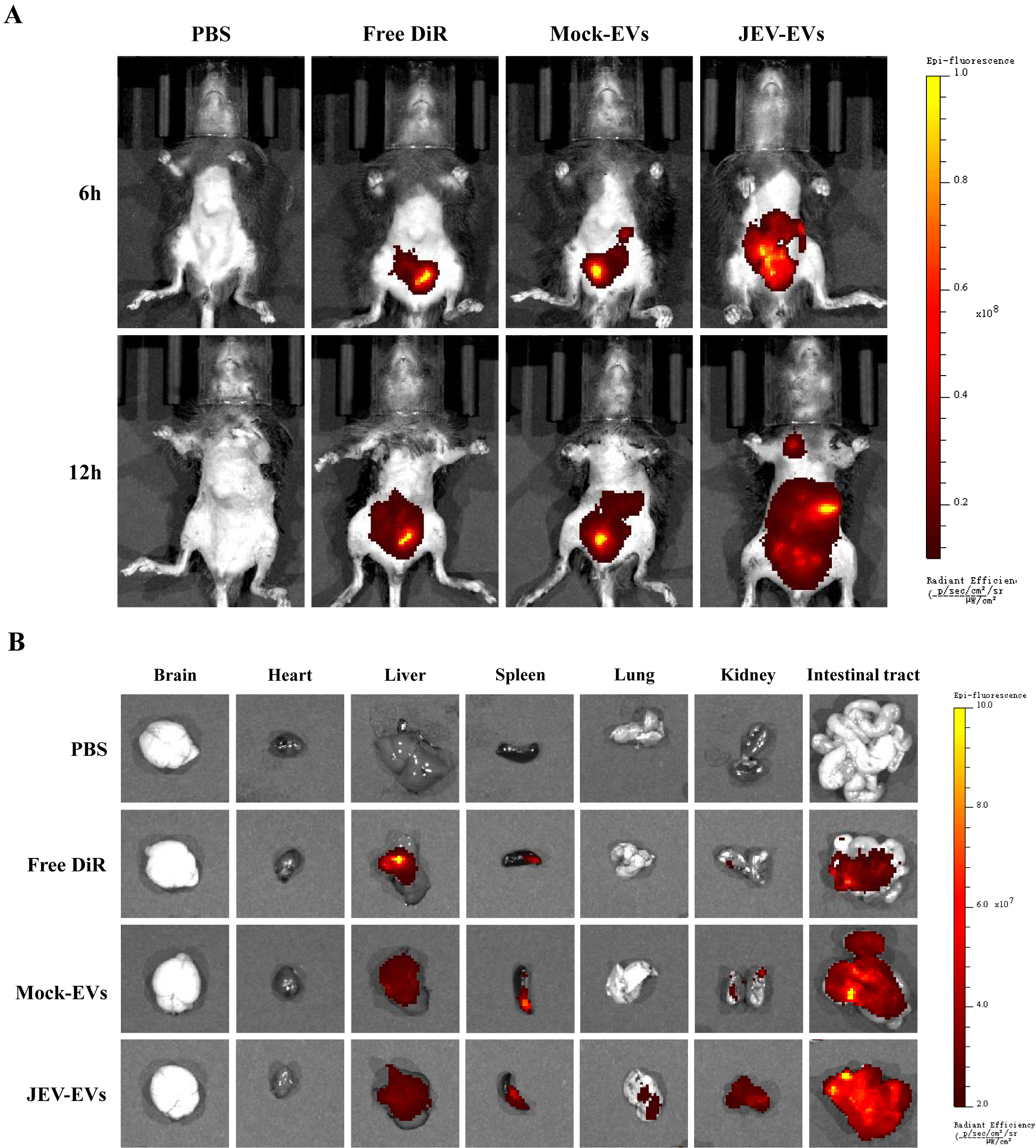

Supplement: Supplementary file 7 — Figure S7. The biodistribution of EVs i.p. in mice. [file JEV2-14-e70033-s007.tif]
