# Supplementary material for: Extracellular vesicles promote the infection and pathogenicity of Japanese encephalitis virus
Source: J Extracell Vesicles. 2025 Jan 9;14(1):e70033. doi: 10.1002/jev2.70033 (PMC11714208; doi:10.1002/jev2.70033)
Supplement: Supplementary file 8 — Supplementary figures. [file JEV2-14-e70033-s001.docx]

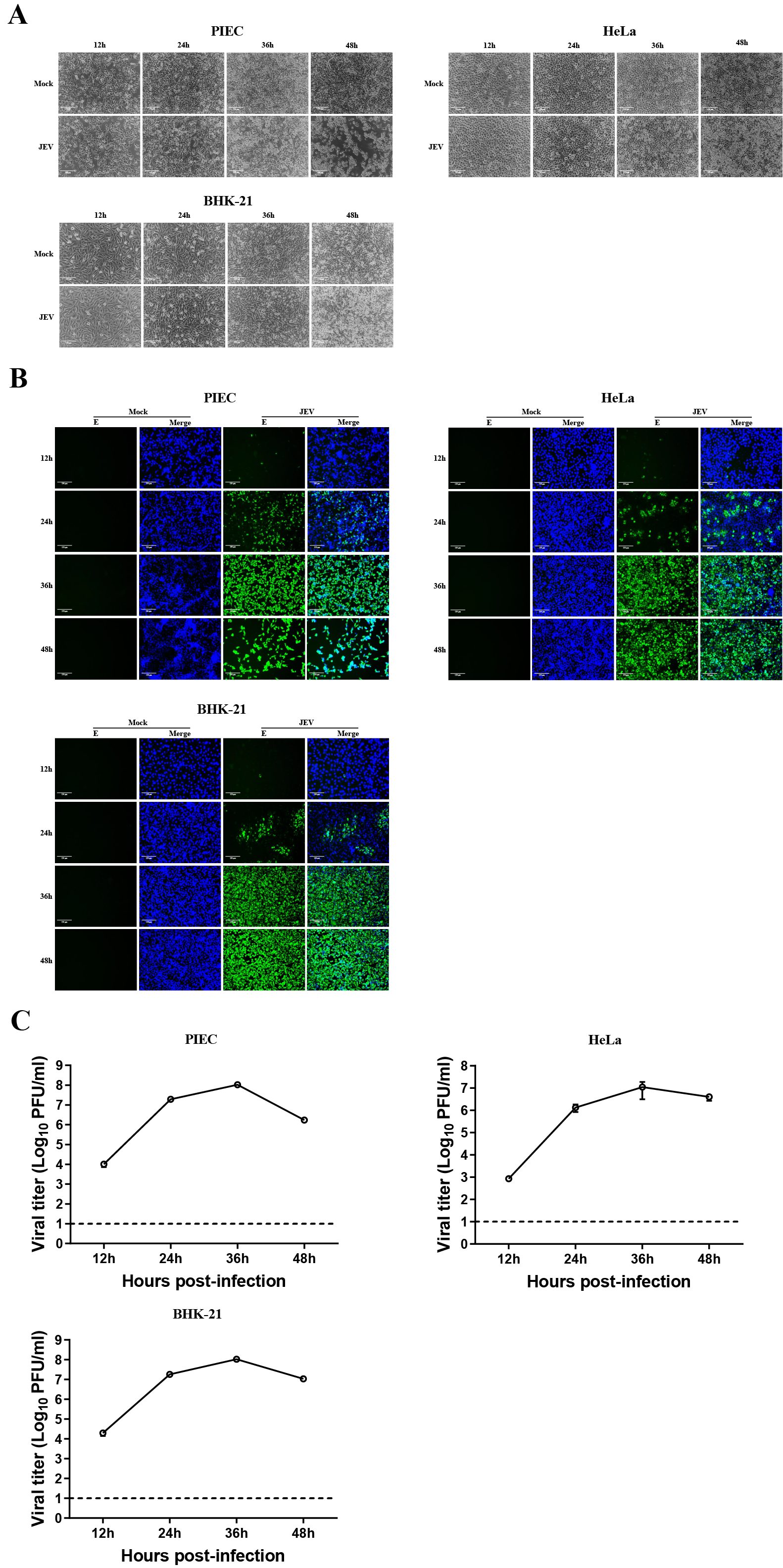


**Figure S1. Conditions for EV isolation from JEV-infected cells.**

PIEC, HeLa, and BHK-21 cells with monolayers were infected with JEV at 1, 1, 0.1 MOI, respectively. After 1 h at 37°C, the supernatants were removed, and the cells were washed three times and replaced with 1% FBS DMEM. The cytopathic effects were observed (A), and the expression of viral E protein (B) and viral titers in the supernatants (C) were evaluated by IFA and plaque assay at different time points, respectively. The representative images from three independent experiments are shown in A and B. Data in C are presented as mean ± SD from three independent experiments. The dotted lines represent the limit of detection. Scale bar: 130 μm.


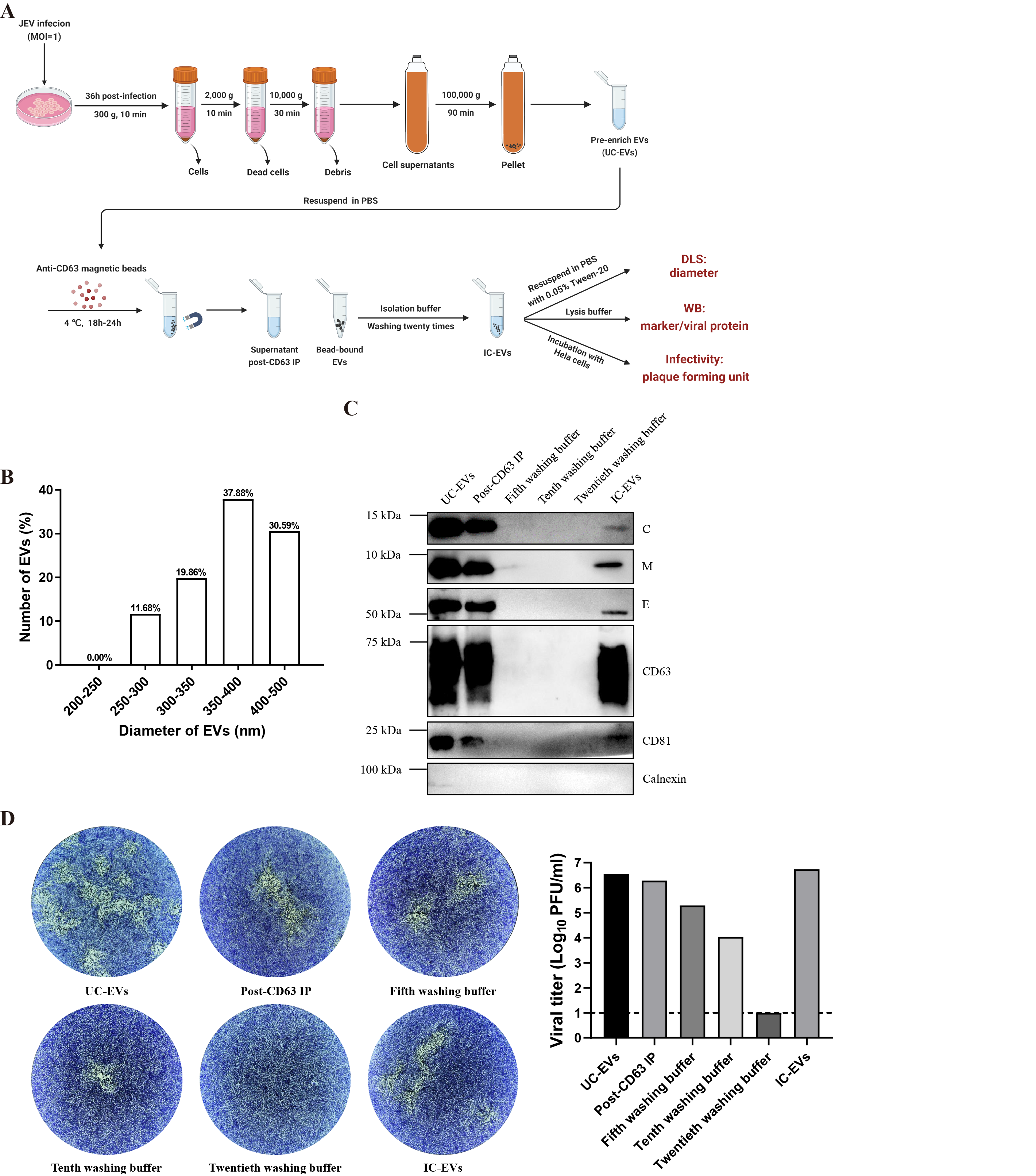


**Figure S2. Purification of EVs from JEV-infected HeLa cells via immunocapture methods.**

(A) Schematic diagram of EVs isolation by ultracentrifugation and further purification by immunocapture. HeLa cells were infected with JEV, and the supernatants were harvested at 36 hpi. The supernatants were centrifugated at 300 g for 10 min, 2000 g for 10 min and 10,000 g for 30 min to deplete cells, dead cells and cell debris, followed by centrifugated 100,000 g for 90 min. The pellets were resuspended in PBS and mixed with anti-CD63 beads. After washing twenty times with the isolation buffer, the supernatants and CD63-EVs-beads (IC-EVs) were harvested and used for DLS, Western blotting, and infectivity. Schematic diagram was created with BioRender (BioRender.com).

(B) The size distribution of IC-EVs. The diameter of IC-EVs was measured by dynamic light scattering. Data are presented as mean value from three experiments. The number of replicates for each measurement varied between 13 and 19, depending on the quality of the samples and the default settings of the dynamic light scattering instrument.

(C) Detection of JEV and EV proteins in IC-EVs. The indicated proteins were detected by Western blotting. The representative blots from three independent experiments were shown.

(D) The infectivity of IC-EVs. IC-EVs were incubated with HeLa cells for 72 h, and the supernatants were harvested and subjected to plaque assay. Dotted line represents the limit of detection. The dotted line represents the limit of detection.


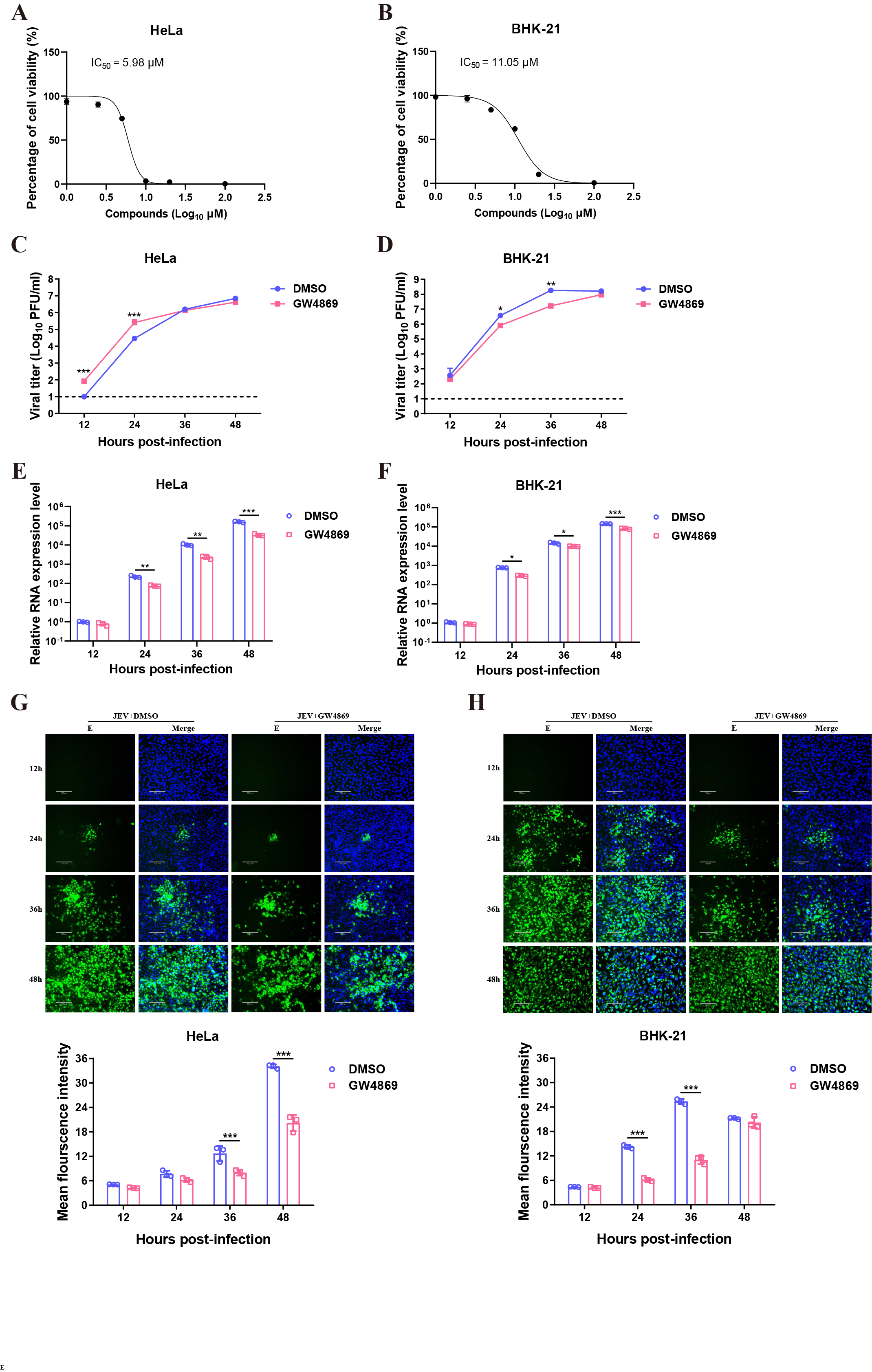


**Figure S3. Impact of EVs on JEV multiplication in HeLa and BHK-21 cells.**

The effect of GW4869 on JEV multiplication. HeLa (A) and BHK-21 (B) cells were incubated with different concentrations of GW4869, and cell viability was measured using Cell Titer-Glo One Solution Assay kit. Subsequently, HeLa (C, E, and G) and BHK-21 (D, F, and H) cells were infected with JEV at a MOI of 0.01, followed by treating with DMSO or GW4869 at a non-toxic concentration at 1 hpi. Supernatants and cells were harvested at the indicated time points, and viral titers in the supernatants (C and D), and viral RNA levels (E and F) and E protein expression (G and H) in the cells were determined using plaque assay, qRT-PCR, and IFA, respectively. Representative images are shown (G and H, upper panels), and fluorescence intensity was calculated using Image-Pro Plus from three visual fields (G and H, lower panels).

Data are presented as mean ± SD from three independent experiments. Statistical analyses were performed using two-way ANOVA test followed by the Sidak post-hoc test (C-H). The dotted lines represent the limit of detection. *, *P＜0.05*; **, *P＜0.01*; ***, *P＜0.001*. Scale bar: 130 μm.


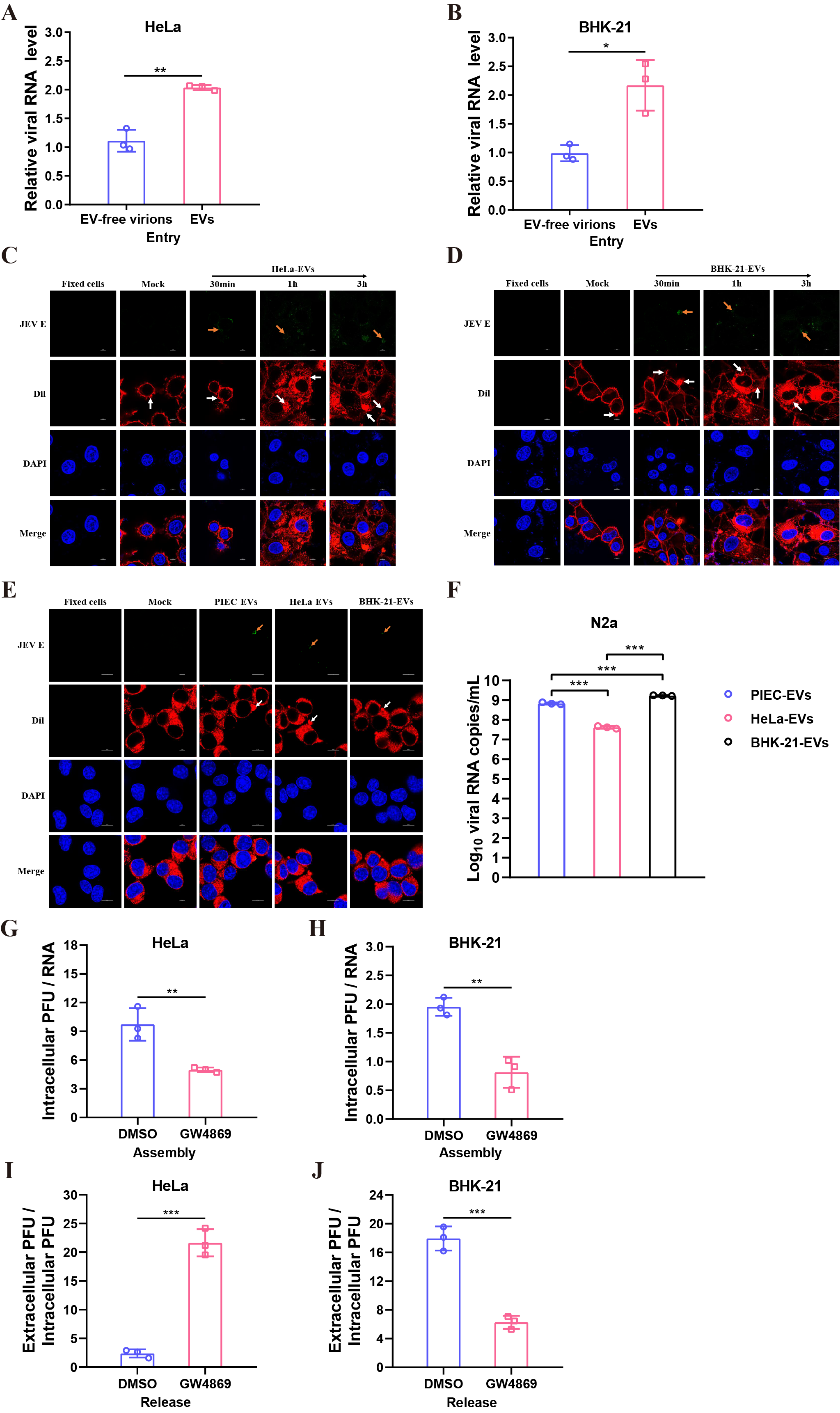


**Figure S4. Impact of EVs on JEV entry, assembly and release in HeLa and BHK-21 cells.**

(A-B) The effect of EVs on viral entry. HeLa (A) and BHK-21 (B) cells were incubated with EVs or EV-free virions containing equal RNA copy number at 4°C for 1 h. After washing with PBS, the cells were further incubated at 37°C for 1 h to initiate viral entry. Then the infected cells were stringently washed three times with PBS and an alkaline high-salt solution to remove surface-associated virus, and the level of viral RNA entering cell was measured by qRT-PCR.

(C-D) Visualization of EVs uptake by host cells. EVs purified from HeLa and BHK-21 cells were labeled with Dil, and these labeled EVs were incubated with HeLa (C) and BHK-21 (D) cells, respectively. The cells were then fixed with paraformaldehyde and blocked with 1% BSA. Subsequently, the cells were incubated with anti-JEV E mAb, followed by an Alexa Fluor 488-conjugated secondary antibody and DAPI. Staining was observed by confocal microscope. EVs and JEV E protein were indicated by white and orange arrows, respectively. Representative images from three independent experiments are shown. Scale bar: 5 μm.

(E) Visualization of EV uptake by N2a cells. EVs purified from PIEC, HeLa, or BHK-21 cells were labeled with Dil, and these labeled EVs were then incubated with N2a cells. Following incubation, the cells were fixed with paraformaldehyde at the indicated time points and blocked with 1% BSA. Subsequently, cells were incubated with anti-JEV E mAb, followed by an Alexa Fluor 488-conjugated secondary antibody and DAPI. Staining was observed using a confocal microscope. EVs and JEV E protein were indicated by white and orange arrows, respectively. Representative images from three independent experiments are shown. Scale bar: 5 μm.

(F) Comparison of the efficiency of EV uptake by N2a cells. N2a cells were inoculated with PIEC, HeLa, or BHK-21 cell-derived EVs containing equal viral RNA copy numbers at 37℃ for 1h. Following the incubation, the cells were harvested, and the viral RNA levels were quantified by qRT-PCR.

(G-J) The effect of EVs on viral assembly and release. HeLa (G and I) and BHK-21 (H and J) cells were infected with JEV at a MOI of 1, followed by treating with DMSO or GW4869 at 1 hpi. The supernatants and cells were harvested at 24 hpi for plaque assay and qRT-PCR, respectively. The ratio of intracellular viral PFU/RNA copies was used to assess the viral assembly efficiency, while the ratio of extracellular PFU/intracellular PFU was employed to evaluate the viral release efficiency.

Data are presented as mean ± SD from three independent experiments. Statistical analyses were performed using two-tailed unpaired Student's t-test (A, B, and G-J), or one-way ANOVA test with Dunnett post-hoc test (F). *, *P＜0.05*; ***, *P＜0.001*.


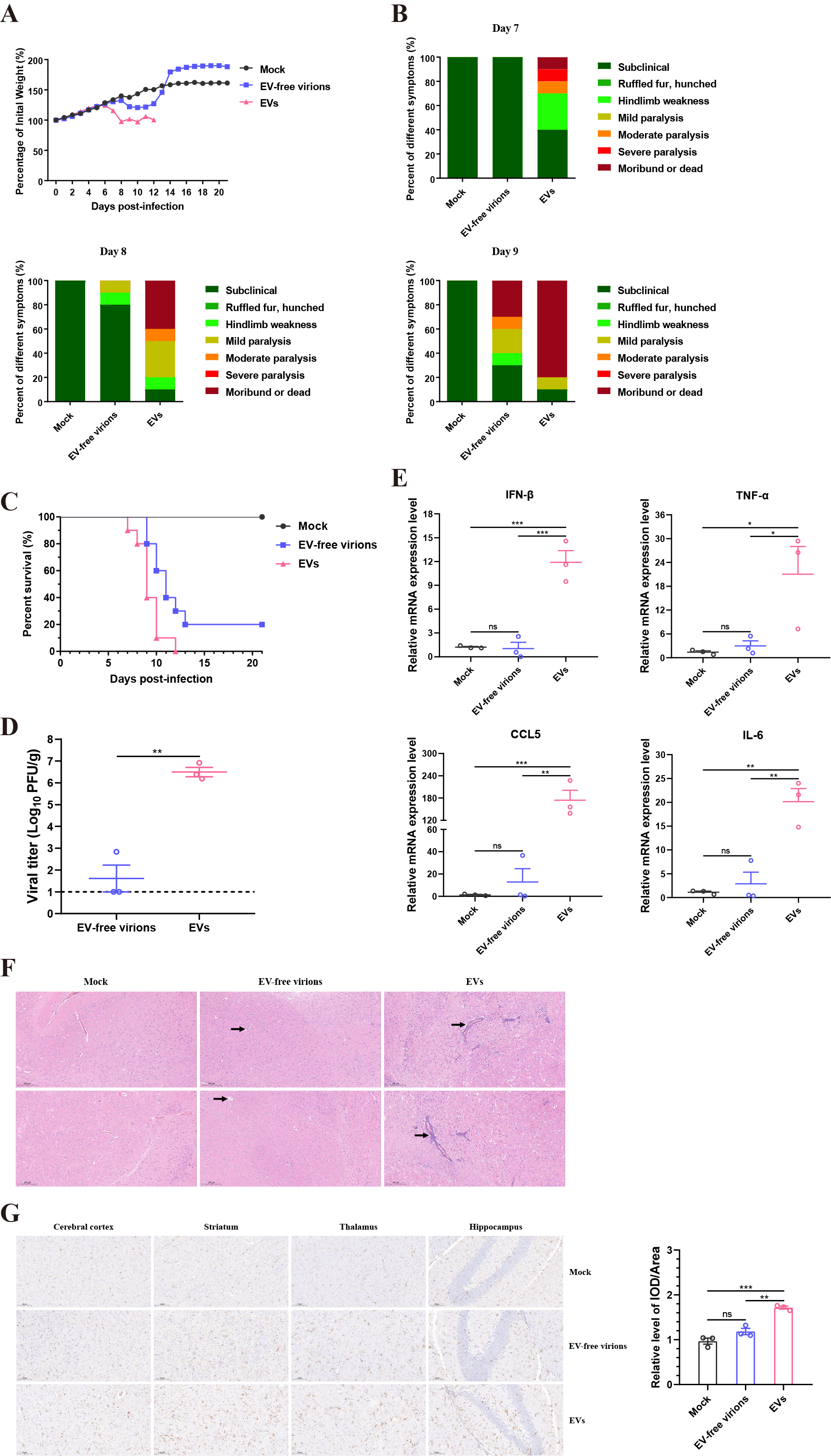


**Figure S5. The effect of EVs on JEV infection in mice via intraperitoneal injection.**

Three-week-old C57/BL6 mice were inoculated with EVs and EV-free virions derived from BHK-21 cells containing equal RNA copy numbers via intraperitoneal injection, respectively. The mice injected with uninfected cell-derived EVs were considered as the mock group. Body weight (A), clinical symptoms (B) and survival rate (C) were monitored (n= 10 mice per group). At 8 dpi, mice from each group were sacrificed, and the viral loads (D) and the mRNA levels of IFN-β, TNF-α, CCL5, IL-6 (E) in the brain tissues were measured by plaque assay and qRT-PCR, respectively (n= 3 mice per group). The pathological changes in the brain tissues were examined by H&E staining, with the vascular cuff indicated by black arrows (F). Immunohistochemistry (IHC) staining of brains was performed to determine the expression of IBA-1 protein, and the integrated option density (IOD) analysis was performed to quantify the staining. Representative images from 3 mice are shown (G, left panel), and the integrated option density (IOD) was calculated from 3 mice with 4 visual fields per mouse using Image-Pro Plus (G, right panel).

The representative images are shown in F and G. Data are presented as mean ± SEM. Statistical analyses were performed using log-rank test (C), or one-way ANOVA test with Dunnett post-hoc test (D, E and G). The dotted line represents the limit of detection. *, *P＜0.05*; **, *P＜0.01*; ***, *P＜0.001*. Scale bar: 200 μm (F), 100 μm (G).


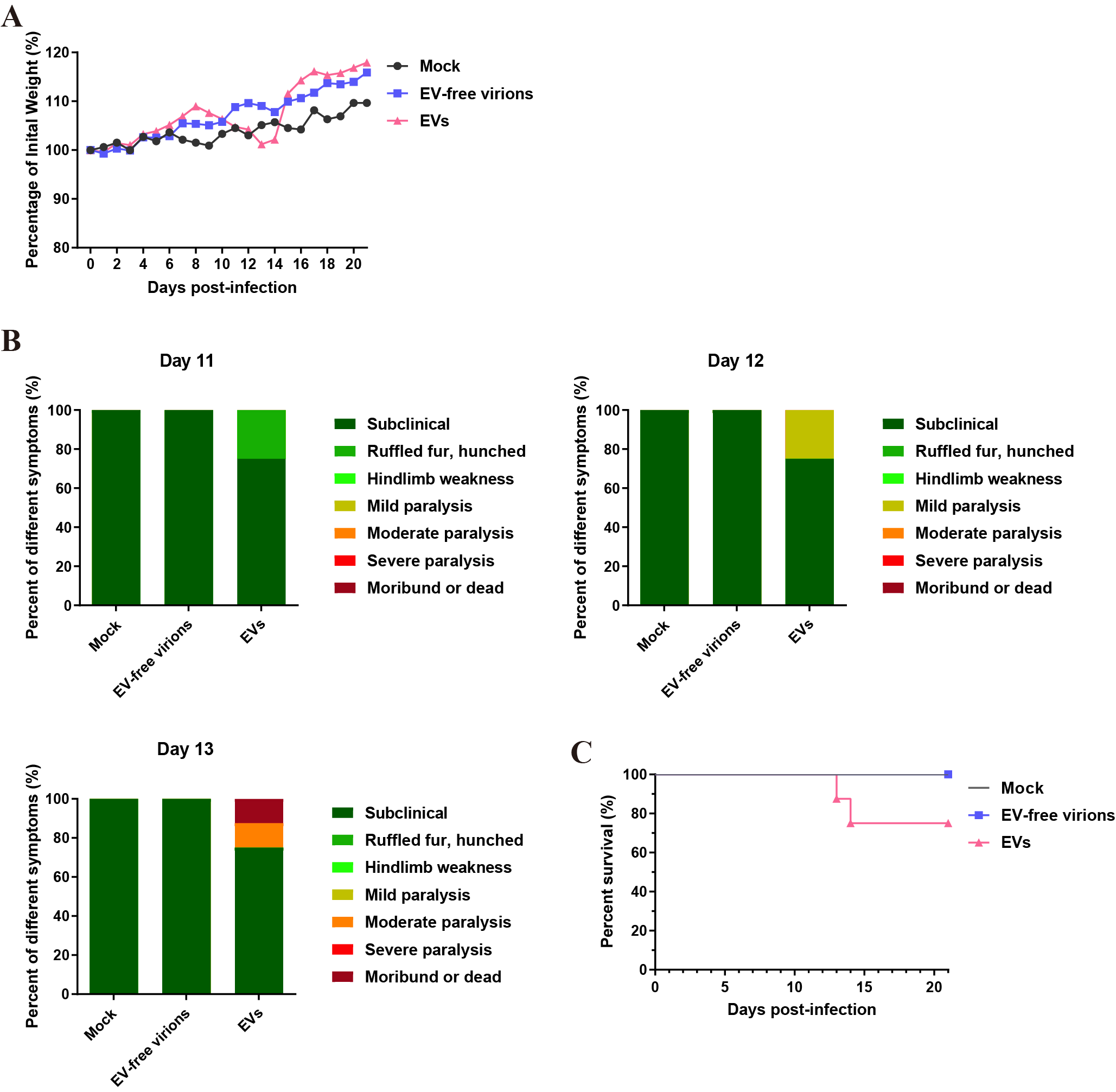


**Figure S6. The effect of PIEC-derived EVs on JEV replication in mice.**

Three-week-old C57/BL6 mice (n=10) were inoculated with EVs and EV-free virions derived from PIEC cells containing equal RNA copy numbers via intraperitoneal injection, respectively. The mice injected with uninfected cell-derived EVs were considered as the mock group. Body weight (A), clinical symptoms (B) and survival rate (C) were monitored and analyzed.


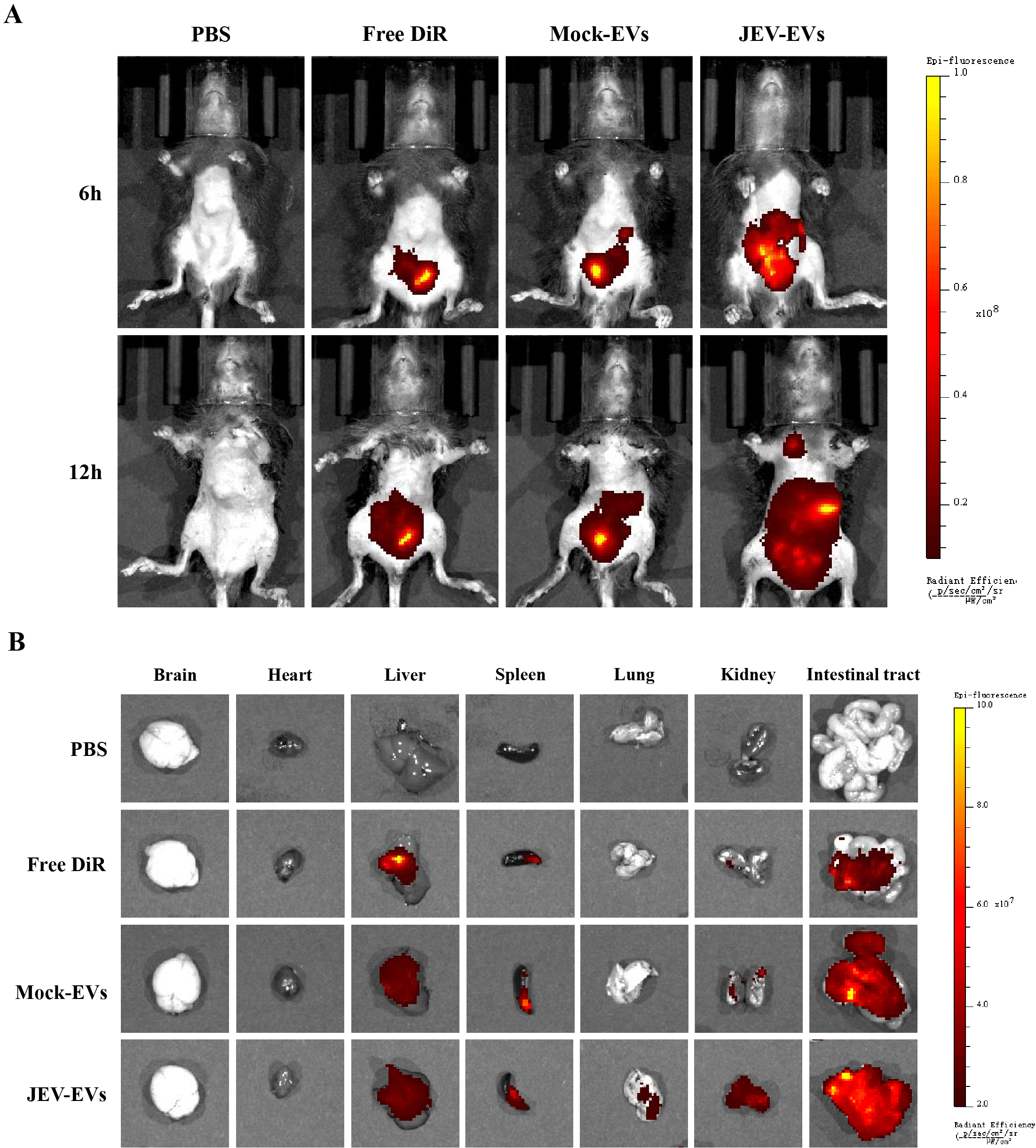


**Figure S7. The biodistribution of EVs injected intraperitoneally in mice.**

EVs derived from JEV or mock-infected BHK-21 cells were labeled with DiR (Mock-EVs and JEV-EVs). These DiR-labeled EVs were then injected intraperitoneally into the mice (n=3 per group). Mice injected with PBS or free DiR were considered as control. The biodistribution of DiR-labeled EVs in the mouse bodies at 6 and 12 hours post injection (hpi) (A) and various organs at 12 hpi (B) was visualized using an *in vivo* optical imaging system (IVIS spectrum, Perkin Elmer), respectively.
